# Joint estimation of survival and dispersal effectively corrects the permanent emigration bias in mark-recapture analyses

**DOI:** 10.1038/s41598-023-32866-0

**Published:** 2023-04-28

**Authors:** Jaume A. Badia-Boher, Joan Real, Joan Lluís Riera, Frederic Bartumeus, Francesc Parés, Josep Maria Bas, Antonio Hernández-Matías

**Affiliations:** 1 Equip de Biologia de la Conservació, Departament de Biologia Evolutiva, Ecologia I Ciències Ambientals and Institut de Recerca de la Biodiversitat (IRBio), Universitat de Barcelona, Av. Diagonal 643, 08028 Barcelona, Spain; 2 Centre for Advanced Studies of Blanes (CEAB-CSIC), Blanes, Spain; 3 Centre for Research on Ecology and Forestry Applications (CREAF), Cerdanyola del Vallès, Barcelona, Spain; 4 Catalan Institute for Research and Advanced Studies (ICREA), Barcelona, Spain; 5 Animal Biology Lab & BioLand, Departament de Ciències Ambientals, Universitat de Girona, Girona, Spain

**Keywords:** Ecological modelling, Population dynamics, Ecology, Zoology, Ecology

## Abstract

Robust and reliable estimates of demographic parameters are essential to understand population dynamics. Natal dispersal is a common process in monitored populations and can cause underestimations of survival and dispersal due to permanent emigration. Here, we present a multistate Bayesian capture-mark-recapture approach based on a joint estimation of natal dispersal kernel and detection probabilities to address biases in survival, dispersal, and related demographic parameters when dispersal information is limited. We implement this approach to long-term data of a threatened population: the Bonelli’s eagle in Catalonia (SW Europe). To assess the method’s performance, we compare demographic estimates structured by sex, age, and breeding status in cases of limited versus large data scales, with those of classical models where dispersal and detection probabilities are estimated separately. Results show substantial corrections of demographic estimates. Natal dispersal and permanent emigration probabilities were larger in females, and consequently, female non-breeder survival showed larger differences between separate and joint estimation models. Moreover, our results suggest that estimates are sensitive to the choice of the dispersal kernel, fat-tailed kernels providing larger values in cases of data limitation. This study provides a general multistate framework to model demographic parameters while correcting permanent emigration biases caused by natal dispersal.

## Introduction

Understanding the drivers of population dynamics is key in basic and applied ecology. Survival is a vital rate that describes the probability of an individual to survive for a given time period and is a major contributor to population growth rate^1^. Dispersal is the movement of individuals from their birth site to their breeding site (natal dispersal) as well as among breeding sites (breeding dispersal)^2^ and has deep implications in population dynamics, from determining emigration to regulating gene flow, inbreeding avoidance, responses to environmental pressures, metapopulation persistence, and source-sink dynamics^3–6^. Consequently, both survival and dispersal processes are central demographic features that carry serious eco-evolutionary implications^1,4^. Thus, obtaining robust and unbiased estimates of both parameters in ecological studies is essential to increase our knowledge about population processes. In addition, this knowledge is crucial to correctly assess the conservation status of populations and design effective case-specific management actions^7,8^, which is essential to guide conservation measures in the urge to alleviate the global biodiversity crisis^9^.

The capture-mark-recapture (CMR) method is widely used in survival estimation^10^. This approach is based on the statistical analysis of monitoring data of marked populations and permits a separate estimation of survival and detection probabilities, which is an essential first step to provide unbiased estimates of survival. In the last decades, analytical advances addressed some limitations and violations of the traditional assumptions of CMR models, such as tag loss^11^, detection and survival heterogeneity^12^, and instantaneous sampling periods^13^. In addition, multistate models allowed for greater flexibility in modelling complex systems, while multievent models enabled dealing with uncertainty in states’ assignments^14^. However, a still problematic limitation of CMR analyses that may lead to considerable biases in demographic parameters is the presence of emigration from the study area, which is a very common process in animal populations^15,16^. If emigration is temporary and random, only detection probabilities become biased^17^. Instead, if emigration is permanent (that is, individuals that leave the study area do not return), models cannot distinguish between emigration and mortality, which leads to an underestimation of survival^18^. Traditionally, this has been accounted for by reporting apparent survival, which is the product of true survival and study area fidelity^10^. In populations where permanent emigration is present, site fidelity is always lower than 1, which makes apparent survival be lower than true survival. In addition, apparent survival has no clear biological meaning, and if estimated with time, sex or age variation, differences in apparent survival among population fractions might correspond to variations in dispersal behaviour and not to true survival differences^18,19^. In the last decades, solutions that estimate survival and site fidelity without explicitly modelling dispersal have been presented^20–22^. However, such designs rely on the availability of additional data, such as observations outside the study area or within sampling occasions, or tag recoveries.

Other alternatives to overcome this bias have focused on explicitly modelling dispersal processes in survival analyses^23^. However, the main challenge in modelling dispersal data obtained from the monitoring of ringed populations is that the resulting dispersal distances generally lead to underestimations of the dispersal kernels of study populations, as some dispersal events (typically the ones ending outside the study area) are rarely detected. Indeed, observed dispersal kernels are a result of the interplay between the true dispersal kernel and detection probabilities, which may be heterogeneous and highly variable across the dispersal range of the species^24,25^. This is due to (1) the border effect, which makes individuals born closer to the limit of the study area more likely to emigrate permanently and therefore remain undetected in subsequent sampling periods^24,26^; and (2) because study areas are finite and limited in respect to the species dispersal capacity, which makes long-distance dispersal events impossible or unlikely to be observed and accounted for in dispersal kernels^8^.

The development of Bayesian hierarchical CMR models (e.g.,^27^) has provided a novel, powerful framework to address these biases^28^. A good example is the Bayesian spatial extension of the Cormack–Jolly–Seber CMR model developed by^26^. This method links the dispersal and observation processes to obtain true survival estimates accounting for emigration probabilities along with corrected dispersal kernels. Nevertheless, spatial CMR models rarely differentiate between natal and breeding dispersal (but see^6^). This apparently subtle difference can have important implications for the robustness of the estimated demographic parameters, as in most species breeding and natal dispersal have different implications for population dynamics^2,8,29^. Generally, natal dispersal distances are larger and involve most or all long-distance movements in a population, which are more likely to fall outside the boundaries of study areas and remain unobserved^5,8,29^. Therefore, natal dispersal is most often the main driver of permanent emigration and may have dramatic effects on the overall dynamics of local populations and metapopulations over broad areas^3^. As a result, kernels of breeding and natal dispersal usually differ in shape, with natal dispersal ones being heavy-tailed and right-skewed^30^. Therefore, if natal dispersal is not explicitly modelled with adequate long-tailed distributions, dispersal kernels may fail to predict unobserved long-distance natal dispersal movements, which may significantly underestimate the dispersal capacity of the species, and consequently, emigration and survival probabilities^8,29,30^. On the other hand, natal dispersal in birds is usually sex-biased, with females usually dispersing farther away than males^31^. In addition, because natal dispersal is intrinsically linked to sexual maturation, dispersal and related processes can show an age trend in long-lived species. Therefore, such processes should be modelled using age and sex structures to avoid biases in demographic estimates.

The aim of this study is to provide a method to minimize the biases in survival and natal dispersal estimations caused by permanent emigration in CMR analyses. For a widely applicable solution to the issue of permanent emigration, it is essential to design a modelling framework that deals with the most common factors that generate this source of bias: (1) natal dispersal, and (2) the restricted sizes of most study areas, which make such movements unlikely or impossible to be detected, and consequently, generate the biases in dispersal and survival estimates. To do so, we perform a joint estimation of natal dispersal and detection probabilities to infer the true natal dispersal kernel, permanent emigration probabilities, and survival estimates in a Bayesian spatial multistate CMR framework^16,24,26,32^. In addition, we model dispersal under two different statistical distributions, i.e., gamma (short-to-fat tailed depending on parameters) and lognormal (heavier and longer tail than gamma), to stress the importance of distribution choice in generating unbiased parameter estimates^8^. To implement this approach, we used as a case study a population of a long-lived territorial bird, the Bonelli’s eagle, located in Catalonia, NE Iberian Peninsula. This study population provides an exceptional scenario to apply the joint estimation approach because: (1) the species shows a very large natal dispersal capacity that can easily exceed the limits of most study areas^33,34^; (2) we carried out an intensive ringing programme over 13 years (ca. 70% of all fledged chicks were tagged in a population holding ca. 6% of the European population); and (3) we benefited from the intensive monitoring of all neighbouring populations of this species, meaning that we could extend our capacity to detect tagged individuals over a broad region covering almost the entire range of this species in Western Europe. In this context, we fitted three different models to assess the performance of our correction method under gamma and lognormal natal dispersal distributions. First, we modelled a scenario in which we included only observations from the study population and performed a classical estimation where detection, survival, and natal dispersal probabilities were not linked for a joint estimation (i.e., “separate estimation”—the SEP-CAT models). Second, we considered only observations from the study population but performed a joint estimation of these parameters (the JOINT-CAT models). Third, we implemented a joint estimation in which all observations of breeding individuals inside and outside the study population were considered (the JOINT-ALL models). This third scenario is expected to provide us the closest approximation to true estimates of survival and natal dispersal kernels, since it is informed by more data on encounters and natal dispersal events over a larger range. Therefore, to assess the effectiveness of the joint estimation method when encounter and natal dispersal data are restricted to the study population, we compared the estimates of JOINT-CAT against those of JOINT-ALL. All models included age and sex structures in survival, recruitment, natal dispersal, and other parameters. Finally, based on the kernels estimated by the models, we performed simulations of natal dispersal movements for each territory and sex to estimate and map continuous probabilities of permanent emigration. The aim of these simulations was to allow a finer-scale evaluation of the sex-specific probabilities of individuals born in each territory to either leave or remain in the study area after natal dispersal.

## Results

Global JMV goodness-of-fit tests indicated an adequate fit to the data for both models with observations from the study area only (CAT; x^2^ = 26.613, df = 26, *p* = 0.430) and models with observations everywhere (ALL; x^2^ = 29.791, df = 28, *p* = 0.373). All 6 models (i.e., SEP-CAT-Lognormal, SEP-CAT-Gamma, JOINT-CAT-Lognormal, JOINT-CAT-Gamma, JOINT-ALL-Lognormal, and JOINT-ALL-Gamma) reached adequate convergence based on the Gelman-Rubin statistic (Rhat ≤ 1.10 for all parameters) and visual inspection of chain mixing^35^.

**Figure 1:**
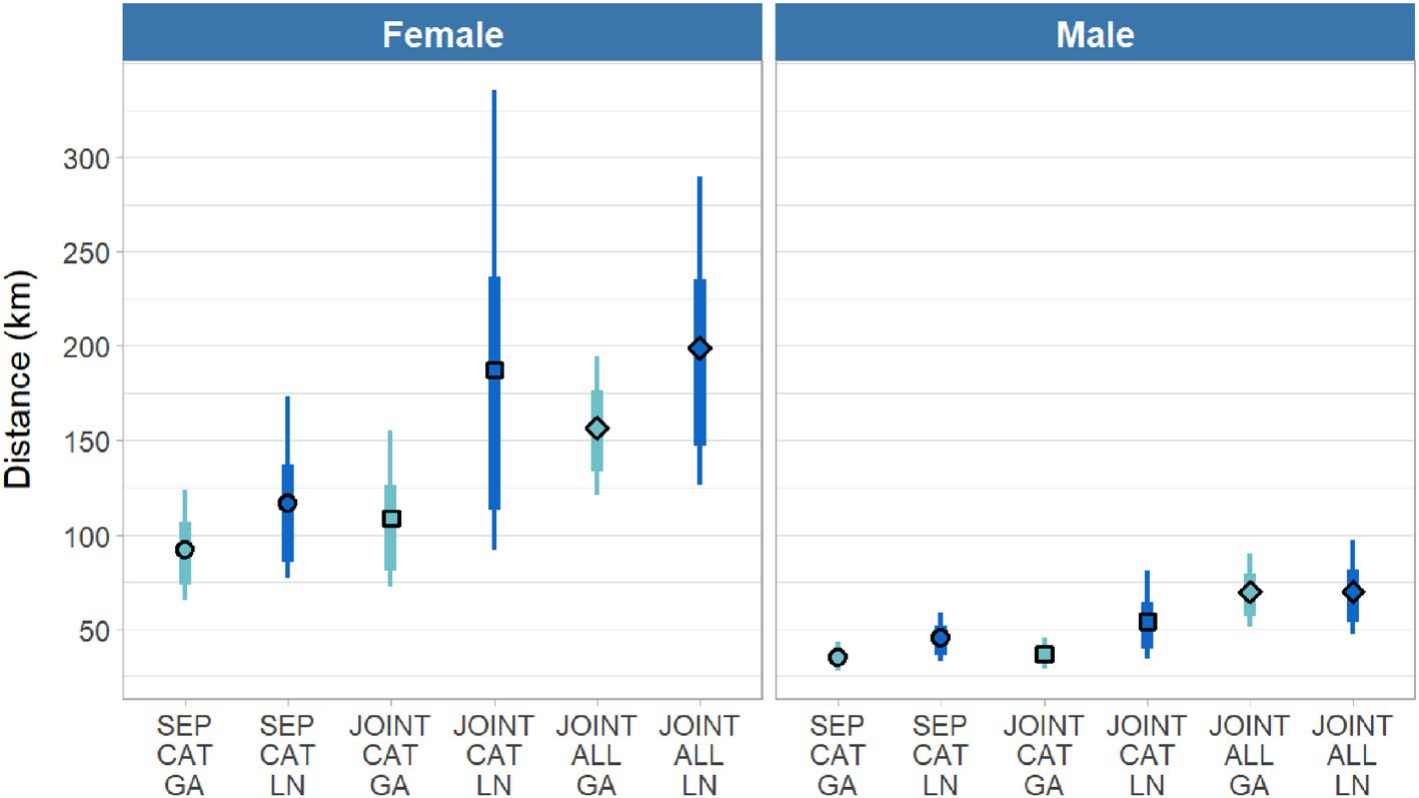
Average natal dispersal distance (km) by model, sex, and distribution (gamma: GA, lognormal: LN). Circles and squares indicate median values. Thick lines indicate the 66% HPDI and fine lines show the 89% HPDI.

**Figure 2:**
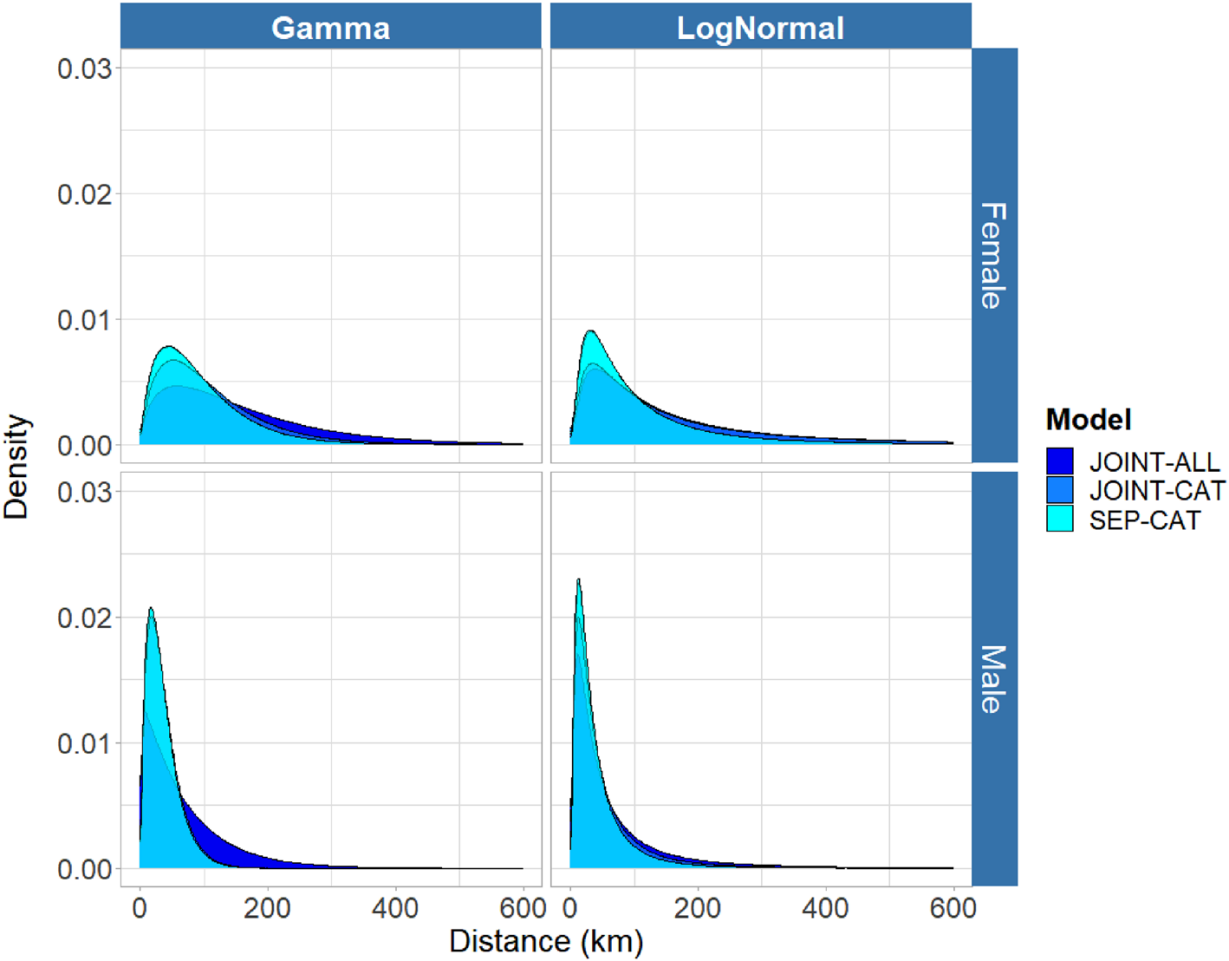
Median natal dispersal kernels (Y: probability density, X: km) by model type, sex, and dispersal distribution.

Natal dispersal estimation showed consistent differences among sexes, with larger distances for females than for males in all scenarios (Tables S1, S2, S3; Figs. 1, 2). Dispersal distances and kernel shapes in JOINT-CAT models approached the estimates provided by JOINT-ALL models and were substantially larger—and considerably more uncertain—than those of SEP-CAT, especially when using lognormal distributions (Fig. 1, Table S2). Female average dispersal distance increased by 16.6 km (89%Highest Posterior Density Interval, HPDI, of the difference = − 34.3–74.2) from SEP-CAT to JOINT-CAT models when using gamma distributions, and by 68.7 km (− 60.2–237.8) in lognormal models. The average dispersal distance of females estimated by JOINT-CAT with lognormal distributions was higher than that of JOINT-ALL when using gamma distributions (30.4 km difference, − 79.8–190.2). In the case of males, distances estimated by JOINT-CAT models increased by 1.6 km (− 10.1–13.3) and 8.9 km (− 19.9–39.5) in gamma and lognormal distributions respectively, compared to SEP-CAT models.

**Figure 3:**
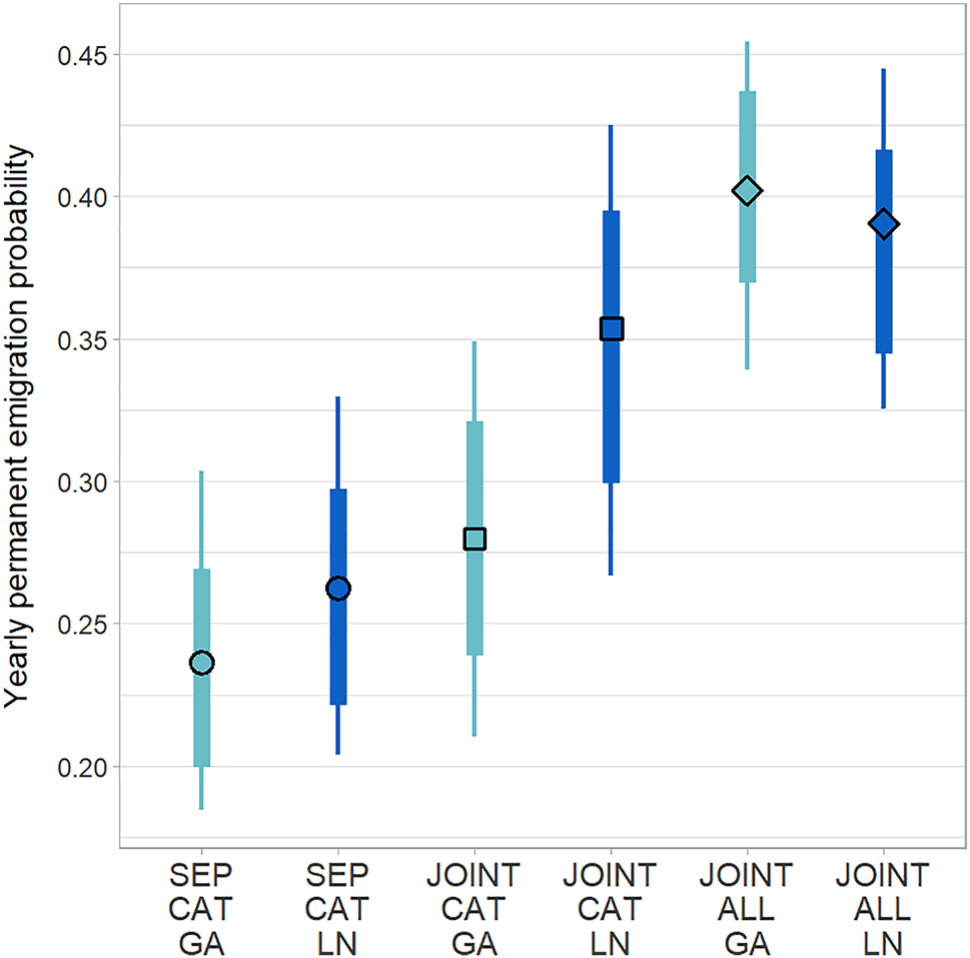
Permanent emigration probability by model and dispersal distribution (gamma: GA, lognormal: LN). Circles and squares indicate median values. Thick lines indicate the 66% HPDI and fine lines show the 89% HPDI.

**Figure 4:**
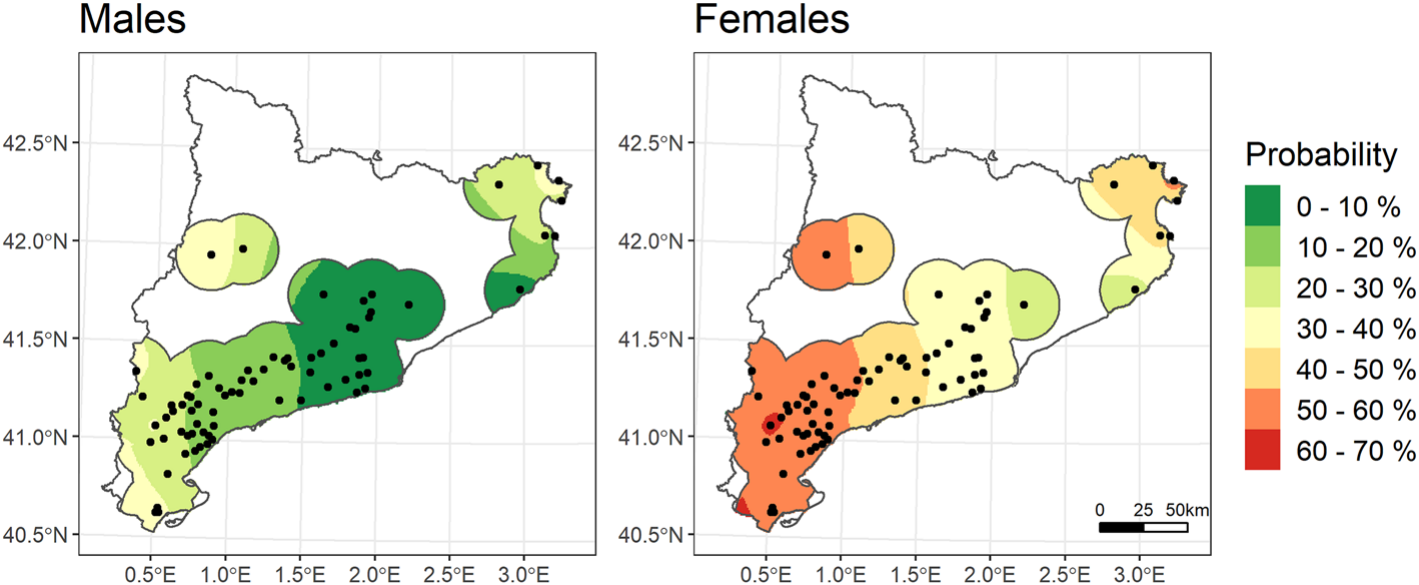
Mean Bonelli’s eagle permanent emigration probabilities mapped by sex following a spatial kriging interpolation across the distribution of the study population. Map source: Natural Earth.

Estimates of permanent emigration probabilities in the population notably increased from SEP-CAT to JOINT-CAT models but fell short of the values estimated by JOINT-ALL models. Lognormal distributions provided closer values between JOINT-CAT and JOINT-ALL estimates (Fig. 3). Using gamma distributions, estimates increased from 0.23 (89% HPDI = 0.18–0.30) in SEP-CAT to 0.28 (0.21–0.35) in JOINT-CAT and 0.40 (0.34–0.45) in JOINT-ALL, while when using lognormal distributions values grew from 0.26 (0.20–0.33) to 0.35 (0.27–0.43) and 0.39 (0.33–0.44) respectively. Mapped permanent emigration probabilities between sexes showed differences by sex and higher chances of leaving the study area closer to its boundaries (Fig. 4). Males born in central areas showed permanent emigration probabilities below 7%, which increased up to 28% in females. In areas closer to boundaries, probabilities peaked to 40% in males and 60% in females.

**Figure 5:**
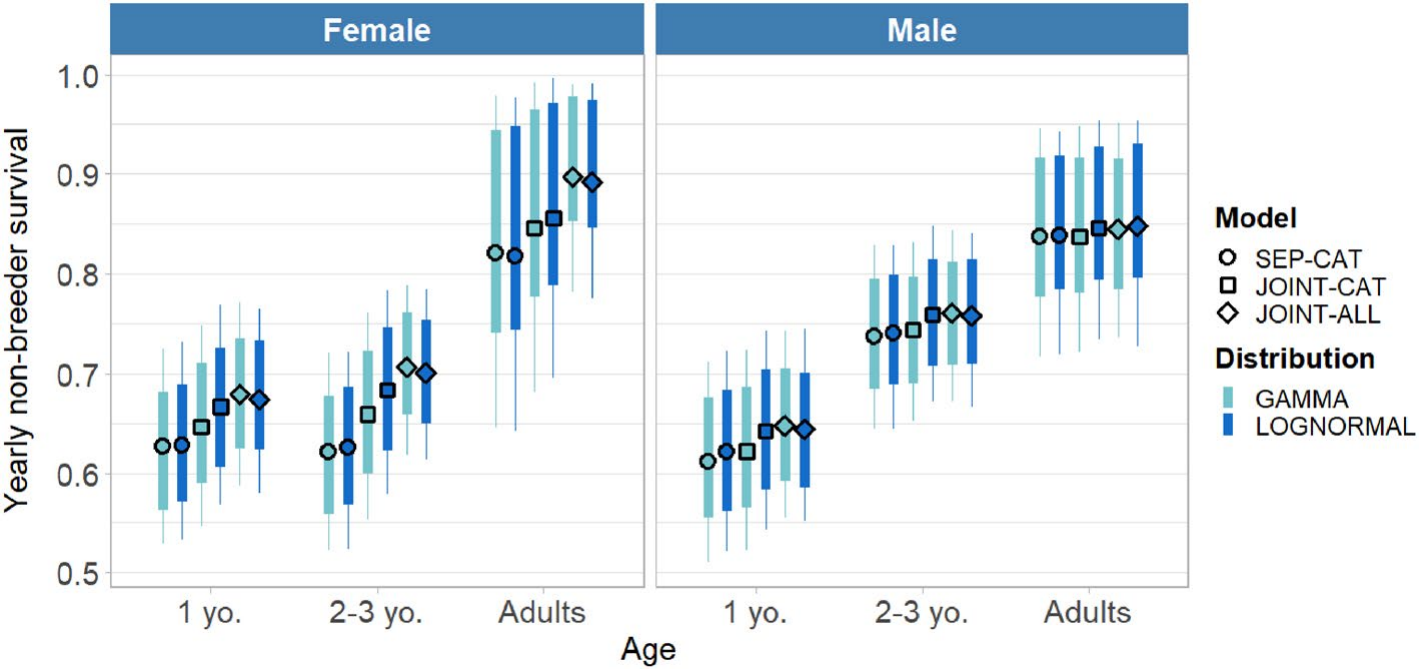
Non-breeder survival by model, age, sex, and distribution. Circles and squares indicate median values. Thick lines indicate the 66% HPDI and fine lines show the 89% HPDI.

Survival estimates of non-breeders showed a clear increase when using joint estimation models, especially in females (Table S1, Fig. 5). Namely, when comparing SEP-CAT and JOINT-CAT lognormal models, female non-breeder survival increased by 0.04 in 1-year-olds (89%HPDI of the difference = − 0.1–0.17), 0.06 in 2 and 3-year-olds (− 0.08–0.2) and 0.04 (− 0.2–0.28) in adults. In males, differences were smaller, with increases of 0.02 (− 0.11–0.15) in 1-year-olds, 0.02 (− 0.12–0.17) in 2 and 3-year-olds, and 0.01 (− 0.15–0.17) in adults. Differences in non-breeder survival between models using gamma and lognormal distributions were especially visible in females. JOINT-CAT lognormal models provided moderately higher values, and closer to those of JOINT-ALL models. On the other hand, survival of breeding birds showed no differences among models and was estimated at 0.87 (0.8–0.94) in females and 0.88 (0.83–0.94) in males (Table S1). Recruitment into the breeding population also showed remarkable differences between separate and joint estimation models in females (Figure S5), while male differences were smaller. In all scenarios, females showed the highest recruitment probabilities for 3-year-olds (JOINT-ALL lognormal model, 0.54; 89%HPDI = 0.39–0.70) and adults (0.41; 0.24–0.58). Male recruitment probabilities peaked in 3-year-olds (JOINT-ALL lognormal, 0.46; 0.31–0.61), but considerably decreased in adults (0.13; 0.06–0.37).

Recapture probabilities were estimated at 0.85 (0.65–0.98) for breeders (P_B_) and 0.045 (0.035–0.056) for non-breeders (P_NB_) in all models. Recovery probabilities of dead non-breeders were estimated very similarly across models as well, with around 0.21 (0.18–0.26). Similarly, in breeders, models estimated recovery at around 0.22 (0.12–0.33). Estimates of the effectiveness of different surveying methods at detecting and identifying individuals in territories, which were calculated to deal with breeder detection heterogeneity and incorporated into first-time recapture probabilities of breeders P_B1_ (see *Methods* and subsection *Recapture parameters* for further details), provided a 0.39 (0.37–0.42) probability of detecting a ring when using conventional territory monitoring, 0.84 (0.81–0.88) when using camera-trapping alone, and 0.989 when using both methods altogether (0.985–0.994).

## Discussion

Survival and dispersal processes are central demographic features of populations^1,4^, though their estimation can be seriously biased by permanent emigration when the dispersal capacities of individuals exceed the dimensions of monitoring areas, a common issue in ecological studies. Here, we present a spatial capture-mark-recapture (CMR) joint estimation approach to model permanent emigration generated by natal dispersal and address subsequent biases in the estimation of dispersal, survival, and other demographic parameters of wild populations. The crucial aspect of this approach is estimating the probabilities of leaving the study area for each marked individual, conditional on their location of birth and natal dispersal distance, and accounting for all modelled sources of uncertainty about the states of the individuals. We applied this method to estimate demographic parameters in a population of a territorial long-lived bird, the Bonelli’s eagle, in which permanent emigration was represented as breeding recruitment outside the study area. We utilized an intensive monitoring campaign that covered the entire range of this species in western Europe to compare the estimates of survival and dispersal kernel under three scenarios: (1) models that formulate a classical separate estimation of data restricted to the focal study area (SEP-CAT models), the most common scenario in monitoring schemes; (2) joint estimation of CMR data and dispersal parameters from the focal study area (JOINT-CAT models); and (3) joint estimation of data from the whole W European distribution (JOINT-ALL models), which is expected to provide us the closest approximation to the true parameter estimates. Our results illustrate that the joint estimation approach provides substantial corrections of survival and dispersal estimates even when only data of the focal study area are considered compared to those of the classical separate estimation approach (Figs. 1, 2, 3, 5).

Our confidence in the effectiveness of the joint estimation approach is based on the fundamental assumption that JOINT-ALL models provide the closest approximation to the true estimates. Indeed, JOINT-ALL models include dispersal and recapture data from the whole range of the Bonelli’s eagle in W Europe. This fact diminishes the effects of censoring on dispersal data: as the study area expands, the chance of observing more dispersal movements and at longer distances increases. In the case of our study, maximum observed natal dispersal distances in the focal study area were 95 km for males and 271 km for females but increased to 500 and 490 km respectively when all data were considered. Hence, the ability of the models that link survival and observation processes to approximate true survival, natal dispersal kernels, and permanent emigration probabilities is enhanced^24,26,32^. Thus, we consider that using JOINT-ALL as a reference model from which to assess the effectiveness of joint estimation methods in cases of limited and truncated data (i.e., JOINT-CAT models) is a robust strategy.

Animal populations are composed of individuals that may show differences in survival and dispersal according to their age, sex, and breeding status. Such intrapopulation heterogeneity may carry important consequences for population dynamics^36^. Our results highlight that the permanent emigration biases may be more intense in specific age classes (Table S1, Fig. 5). In addition, we found that survival estimates of non-breeders are sensitive to the permanent emigration bias. This fact should be expected for many animal species, as non-breeders often undertake the longest movements in their lives (natal dispersal) right before first breeding. Non-breeding fractions of populations have a key role in population regulation and stability, and therefore biased survival estimates may lead to inaccurate assessments of the status and prospects of whole animal populations^37^. Specifically, Hernández-Matías et al.^38^ showed that a survival increase of ca. 8% in non-breeders would turn our study population from decreasing to self-sustaining. According to our results, survival differences of such magnitude can be generated by permanent emigration biases, especially in females. Indeed, female survival was systematically higher in JOINT-CAT compared to SEP-CAT models, differing by 0.04 in 1-year-olds, 0.06 in 2 and 3-year-olds and 0.04 in adults when using lognormal models, and differences with JOINT-ALL models are around 0.08 in 2 and 3-year-olds and adults. In this line, our results showed more intense survival biases in females than in males, which is related to the fact that females show larger natal dispersal and thus higher propensity to permanent emigration. Sex differences in natal dispersal are common in wild populations^39^. Thus, if demographic parameters are estimated without considering permanent emigration and sex-structured dispersal, the resulting sex differences in apparent survival estimates may actually be signalling differences in dispersal behaviour and site fidelity rather than true survival differences^19^. Furthermore, our results show that permanent emigration biases can also affect estimates of the age of sexual maturity (i.e., recruitment in this study). Unbiased estimates of sexual maturity are essential in models of population viability, as population growth rates may be very sensitive to them^40^. In summary, our findings suggest that the potential of permanent emigration to cause bias in ecological studies, as well as population and species assessments, should not be neglected. Hence, study designs accounting for permanent emigration should be implemented in demographic studies whenever possible, especially in species with long dispersal capacities. It is true that bias correction in demographic parameters may not be complete in cases of limited data as in the JOINT-CAT scenario (Table S1). However, substantial bias reductions as the ones found here may help improving the accuracy of subsequent analyses based on these estimates, such as population viability analyses, integrated population models or assessments of conservation status.

Dispersal has deep implications in eco-evolutionary processes^5,6^. However, long-distance dispersal movements are difficult to detect in many study systems, and therefore, obtaining unbiased estimates of dispersal kernels may be challenging. Interestingly, our findings emphasize that the method presented here is sensitive to the choice of the dispersal distribution. In the case of JOINT-ALL models, results are almost identical when using gamma and lognormal distributions. This suggests that whenever larger amounts of data are available from a wider geographical range, both distributions provide very similar corrections of demographic parameters, even if the shapes of the dispersal kernels differ (Tables S1, S2, S3; Figs. 2, 3). However, our results show that when the dispersal data are strongly truncated as a result of only having data from the focal study area available (i.e., the most common scenario in ecological studies, represented by SEP-CAT and JOINT-CAT), joint-estimation models (JOINT-CAT) provide closer estimates of all demographic estimates to JOINT-ALL when lognormal distributions are used. This effect is particularly visible in average natal dispersal and permanent emigration estimates, which are considerably higher in JOINT-CAT lognormal than in JOINT-CAT gamma, and notably closer to JOINT-ALL estimates. A reasonable explanation is that lognormal distributions are heavy-tailed and thus may provide larger probabilities for long-distance dispersal movements even when these have been poorly detected^8,25,29^. This rationale would match the recent findings of Fandos et al.^41^, who found that long-distance dispersal movements were frequent across bird species, and dispersal kernels were generally better represented by heavy-tailed distributions. This suggests that heavy-tailed distributions can generally be more adequate when modelling natal dispersal in joint estimation formulations. However, given that in many study cases there may be little to no information about the shape and the tail-end of the true natal dispersal kernel, providing model results under different dispersal distributions may be a more conservative choice^3,8,29,41^. In ideal cases where unbiased dispersal data are available (i.e., telemetry or different dispersal estimation methods), these should be primarily considered for the choice of adequate natal dispersal distributions.

For a widely applicable solution to the issue of permanent emigration, it is essential that our modelling framework is easily extrapolated to other study systems. In basic terms, our modelling approach consists of a multistate CMR submodel and a spatial submodel appropriately linked to infer permanent emigration probabilities. Here, we incorporated some modelling specificities to account for the particularities of our study system such as territorial behaviour, delayed maturity, detection heterogeneity in breeders, or the possibility to encounter dead individuals. However, all these characteristics can be easily modified to handle a wide range of multistate formulations either simpler or more complex, taking advantage of the wide flexibility of Bayesian hierarchical models (see Appendix S3 and Figures S6 to S11 therein, where we present a brief guide to adapt the joint estimation formulation to other study designs, starting by the simplest possible designs and moving to examples without detection heterogeneity and dead recoveries). The Joint Estimation approach may be useful for species where individuals are tagged at the natal site, and for which a fraction of all dispersing individuals may disperse outside the study area. A key point is to be able to model a species or population distribution range to the extent it encompasses the dispersal capacity of the population. In cases where the range is not known, multiple methods are available to estimate it^42^. In our example, territory locations were known with certainty or simulated following distribution and census data. In territorial species where territory locations cannot be estimated, or in species whose space use is not structured into territories, space may be divided into grids with detection probabilities assigned depending on their location (i.e., inside or outside a study area;^26^). Isotropical dispersal kernels are often assumed, but many other options can be implemented in a Bayesian hierarchical framework. For instance, dispersal may be modelled using a longitude and a latitude component^26^ or considering individual heterogeneity in dispersal behaviour^43^.

The flexibility of Bayesian models is further illustrated by the possibility of mapping emigration probabilities in our study area (Fig. 4). The notable differences found among sexes and across space can provide a deep understanding of population and metapopulation dynamics to (1) illustrate the magnitude of the border effect and how it can differentially affect different fractions of a population; (2) reveal the heterogeneous contribution of territories to local population processes; and (3) understand whether unobserved individuals might be more likely attributable to either emigration or mortality. Such knowledge can be important to managers as often critical conservation decisions have to be made within short periods of time and with few up-to-date information^44^.

Despite the generality and flexibility of our modelling approach, there may be situations where the joint estimation is not adequate. The present framework has been designed to model natal dispersal and subsequent permanent emigration, since in most animal species natal dispersal accounts for most-to-all long-distance dispersal movements in a population, and is by far the most important determinant of permanent emigration. This view is often considered the paradigm in animal populations^2,45^. However, the method does not account for breeding dispersal. This type of dispersal is commonly composed of short movements that mostly generate cases of not permanent but temporary emigration, which may mainly bias recapture probabilities and often has a more limited effect on other demographic parameters^17^. Nevertheless, there may be some species or populations in which breeding dispersal is similar or larger than natal dispersal. In such cases, breeding dispersal may (1) become a significant contributor to permanent emigration, and (2) frequently cause individuals that have moved outside the study area due to natal dispersal to come back to it. Both phenomena, if frequent, may have the capacity to bias the estimates provided by the joint estimation method, as there are sources of permanent emigration that are not accounted for, or on the contrary, permanent emigration may be confused with temporary emigration. Large breeding dispersal patterns appear to be related to specific ecological conditions rather than evolutionary or phylogenetic reasons^2^. In birds, this may be the case for populations with highly patchy distributions and very specific ecological requirements, like wetlands that may be poorly connected^46^. Thus, the present method should be used with caution when considerable permanent emigration caused by breeding dispersal is suspected, and other approaches may be more suitable (e.g.,^6,26,43^). One further limitation of the joint estimation approach may emerge in cases of study areas very restricted in size combined with populations with large natal dispersal capacities. In such cases, very few dispersal events may be detected, which may hamper the ability of joint estimation methods to provide consistent corrections in demographic parameters^26^. To address this issue, Bayesian models might provide a solution in the form of priors if direct or indirect information about dispersal exists^47^. In these situations, telemetry data could be a very useful source of dispersal information, if available. Other potential limitations of the joint estimation approach may arise from the fact that the method requires modelling territories or the distribution of the species inside, and especially outside the study area. In some species, the information about the distribution or number of territories can be poor. In these cases, modelling wrong territory distributions or numbers along space could push the estimates of dispersal and permanent emigration probabilities to be either underestimated or overestimated. These biases could propagate and lead to unrealistic estimations of true survival and other demographic parameters. Hence, a realistic modelling of the range, number, and/or distribution of territories appears important for an optimal performance of the method.

Overall, the joint estimation method presented here provides a promising framework to reduce biases in dispersal and survival estimates in mark-recapture analyses. In addition, this may be a useful formulation for future studies to assess additional sources of individual or population heterogeneity in dispersal and permanent emigration as well as their impacts in survival and other demographic parameters. Here, we show how the permanent emigration bias has different effects in the estimates of males and females. However, age, morphologic, and genetic traits may also affect individual permanent emigration probabilities^2,6,48,49^. Breeding density, nest occupation, and intraspecific competition in source and destination areas may also contribute to shaping natal dispersal, with higher densities or interactions often associated with larger dispersal patterns^15^. Including information on such processes (e.g., as covariates) in future joint-estimation-based studies may help disentangling the complexity of dispersal processes and their multiple population and evolutionary drivers using mark-recapture data.

## Methods

### General modelling approach

**Figure 6:**
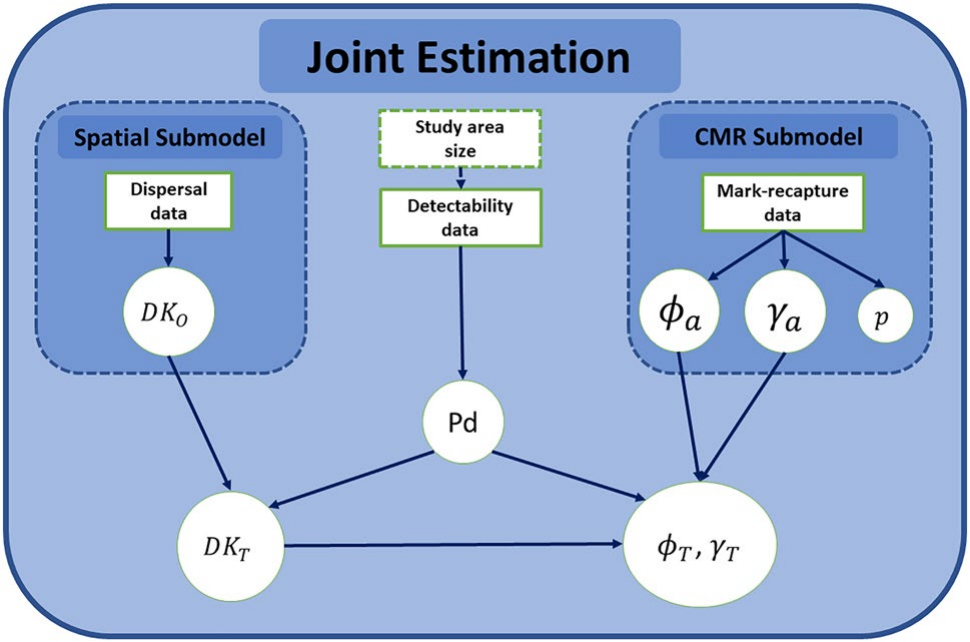
Diagram of the Joint Estimation framework. Data types are inside solid squares (study area size is inside a dashed square as study area information is implicitly provided by detectability data). Parameters are shown inside circles. Parameters DK_o_ and DK_T_ stand for the observed and the true dispersal kernel respectively. Parameters φ, γ, and *p* from the CMR submodel account for apparent survival, apparent recruitment, and recapture estimates. Parameter P_d_ provides information on detectability at different dispersal distances for every individual and links the spatial and the CMR submodels. Note that the absence of parameter P_d_ would lead to a separate (i.e., unlinked) estimation of survival and dispersal.

The joint estimation approach described here consists of a Bayesian hierarchical model structured in a multistate CMR submodel and a spatial submodel (Fig. 6). Both components must be linked by a parameter that informs the model about permanent emigration probabilities by means of estimating an individual’s probability to permanently leave the study area conditional on its natal dispersal distance, in addition to mortality considerations. We use the spatial/dispersal information provided by this parameter to guide a state transition in the multistate CMR submodel, in which an individual may move from a live state at *t* to an absorbing state (a permanently unobservable state) at *t* + *1* in case it leaves the study area. This transition is the key point of the joint estimation approach, as it allows the model to infer the true dispersal kernel and subsequently address the biases in demographic parameters. We provide a detailed description of the mathematical, statistical, and modelling features of the joint estimation approach in the Methods section *Joint Estimation of natal dispersal kernel and detection probabilities*. This is a flexible formulation that can be applied to a wide range of species and ecological contexts. Overall, it is applicable to species in which natal dispersal is the main driver of permanent emigration and shows markedly larger distances than breeding dispersal, as is common in most wild populations^2^. In addition, study areas should not be very restricted in size, to the extent that breeding dispersal does not easily exceed its limits and thus contribute to permanent emigration along with natal dispersal.

As estimating natal dispersal is a central point of the method, it is essential that monitoring schemes focus on tagging individuals at birth sites and reencountering them as breeders. Since natal dispersal is intrinsically related to sexual maturity, the transition matrices of multistate CMR models should be adjusted to reflect the breeding biology of the study species (i.e., modelling maturity, or the age of first breeding as a fixed or varying age;^50^). Here, we adapted this formulation to the Bonelli’s eagle study system, in which the study area was structured into territories. We modelled recruitment to a breeding territory as an equivalent to sexual maturation since individuals start showing territorial behaviour right after maturation. In addition, in our example, whenever an individual recruits to a non-monitored territory (i.e., outside the study area), it is counted as a permanent emigration case. We included dead recoveries to increase the precision of our estimates, but these are not necessary to implement this design (Appendix S3). Further insights on the applicability and potential shortcomings of this approach are developed in the Discussion section.

### Study population and life cycle

The Bonelli’s eagle is a territorial long-lived raptor with delayed maturity and low breeding rates whose range extends from south-east Asia to the western Mediterranean. Our focal population is located in Catalonia (NE Spain) and holds ca. 82 breeding pairs^51^. As in many other territorial raptors, the population is structured into the non-territorial (i.e., non-breeding) and territorial (i.e., breeding) fractions. After the post-fledging dependence period, individuals are non-territorial and show a transient nomadic behaviour with frequent visits to areas away from breeding territories^33^. Birds become territorial (i.e., breeders) after sexual maturity and recruitment to the breeding population, which mostly occur between three and four years of age^34^. As they establish in territories, birds start displaying territorial and pair-binding behaviour, with strong fidelity to their first breeding areas and very infrequent breeding dispersal^52^. Between breeding seasons, individuals either do not leave their territories or stay in their surroundings^33^. Given the strong link between sexual maturity, territoriality, and breeding in this species, we refer to (non)-territorial and (non)-breeding individuals as equivalent terms.

We used data from an intensive tagging and monitoring programme (2008–2020), where 461 chicks born in 51 different territories were ringed in their nests with metal and alphanumeric colour rings. All rings were riveted to avoid tag loss. To recontact tagged non-breeders, we monitored the two main dispersal areas away from breeding territories in Catalonia, which are located in Tarragona and the Lleida Plains. Dispersal areas are sites with large prey availabilities, which attract large amounts of non-breeders^33^. In addition, we monitored 76 different territories in our focal study area to detect breeding birds. For territory monitoring, we used both telescope observation routines and camera-trapping monitoring from January to May (breeding season). 178 marked individuals were recontacted alive, 83 of which were territorials (60 inside the study area, 23 outside). In addition, we recovered 75 dead marked individuals (62 non-breeders, 13 breeders) from the surveillance of territories, dispersal areas^33^, and zones of high mortality risk (i.e., power infrastructures, ponds) by monitoring and ranger teams. Sexing was done by DNA analyses, biometry^53^ and breeding behaviour assessments. Given the strong breeding site fidelity of territorial raptors, monitoring schemes usually prioritize breeding territories that were known to be occupied in previous years, while recruitments in unoccupied territories might become unnoticed. In addition, whenever a ringed individual is detected in a territory for the first time, monitoring teams usually put additional efforts in reading the rings from year to year. While this is a common monitoring strategy, it may generate heterogeneity between the first-time and subsequent detection probabilities of breeders. Hence, this source of breeder detection heterogeneity needs to be modelled accordingly to obtain unbiased demographic estimates^50^.

### The multistate CMR submodel

#### Multistate modelling

We modelled three different scenarios (SEP-CAT, JOINT-CAT and JOINT-ALL) under gamma and lognormal dispersal distributions. We used Bayesian multistate CMR designs to model survival, dispersal, and territory recruitment, along with recapture and recovery probabilities. Models were built and run in the BUGS language using the R package NIMBLE^54^. For the modelling of multistate transitions, we used the same state-transition matrices and age structures across models following the extensive knowledge about the demography of the species^55^ to facilitate result comparisons among models. We chose vague priors for all but dispersal variables (i.e., Uniform (0,1) and Beta (1,1) for variables bounded between 0 and 1; Normal (0, sd = 1.5) for logit-transformed variables;^50,56^. See section *The Spatial Submodel* for information about dispersal priors. In addition, to avoid violation of the instantaneous sampling assumption, we pooled sampling occasions into 6-month periods: January to June, and July to December^10^. As in most Bayesian CMR models, we used a hierarchical state-space design with two components: first, the state process described by the state transition matrix, which defines how individuals change their biological states between consecutive capture occasions; and second, the observation process described by the observation matrix, which indicates how individual observations relate to the states of the individuals. Transitions among true states are represented by a matrix of latent states *z*, while information about observations is provided by the CMR matrix *y*. Transitions among states and associations between states and observations were modelled with a categorical distribution

$${z}_{ind, time} \sim Categorical(p{s[z}_{ind, time-1}])$$

where *ps* is the matrix of transitions among states (Appendix S1, Figure S1), and

$${y}_{ind, time} \sim Categorical(p{o[z}_{ind, time}])$$

where *po* is the event matrix (Figure S2).

#### The state and observation processes

**Figure 7:**
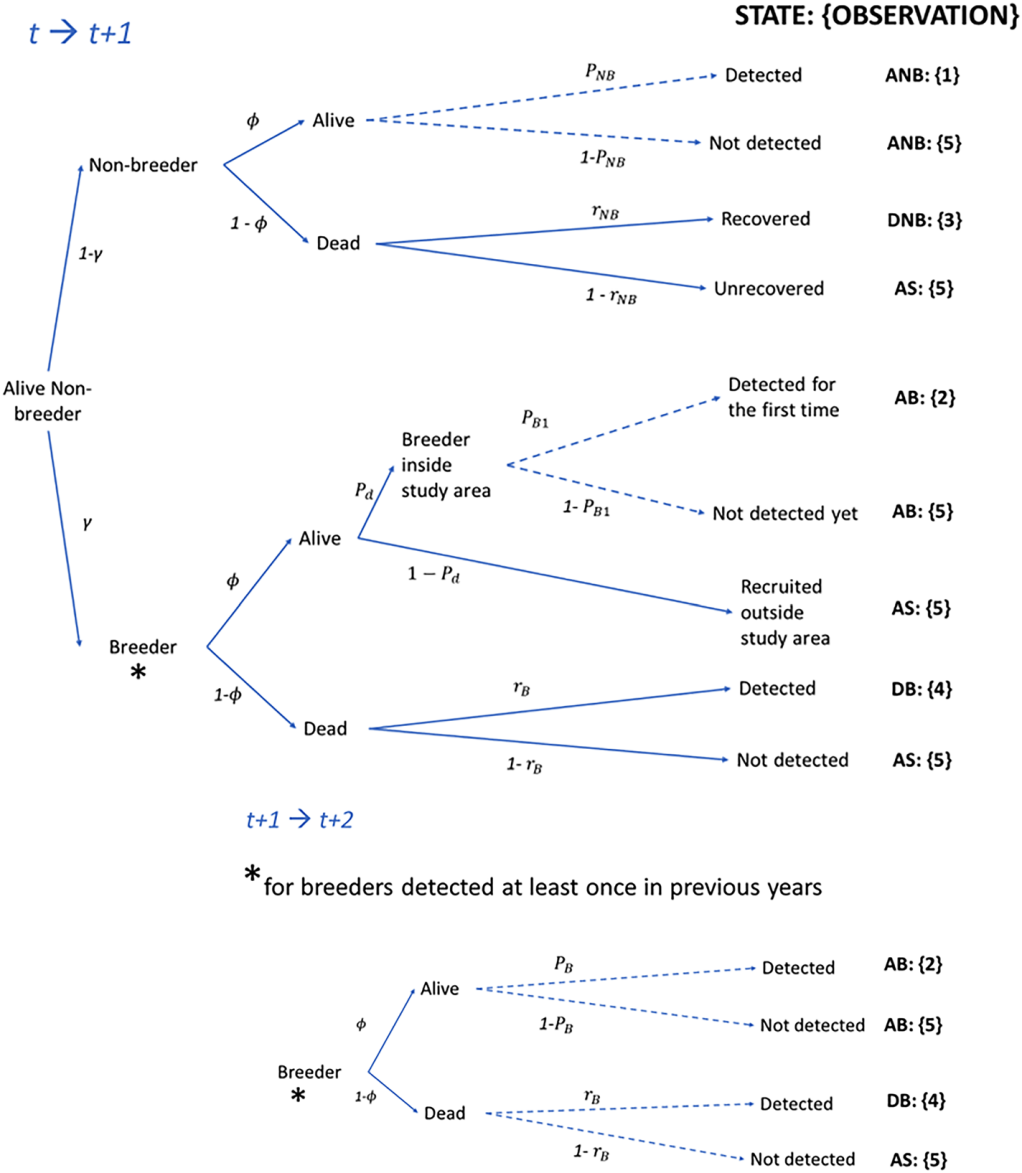
Graphical representation of state transitions, observations, and parameters from occasion *t* to *t* + *1* for an individual ringed at birth. Solid lines indicate transitions in the state matrix and dashed lines indicate transitions in the observation matrix. State codes are ANB (Alive Non-Breeder), AB (Alive Breeder ), DNB (Dead Non-Breeder), DB (Dead Breeder) and AS (Absorbing State). Numbers in brackets ({}) indicate corresponding codes in the observation matrix columns and encounter histories. Parameter symbols stand for survival (ϕ), recruitment (γ), recapture (*P*_B_ and P_NB_), recovery (*r*) and the first-encounter breeder recapture parameter (P_B1_). *In the lower diagram, transitions of breeding individuals from *t* + *1* to *t* + *2* are shown. Because of the implementation of breeder recapture heterogeneity, individuals that become breeders at t + 1 may or may not be observed for the first time depending on probability P_B1_. Once individuals are first observed, the observation probability P_B_ is implemented at subsequent occasions.

We defined five different states common to all models (Figure S1): (1) Alive Non-Breeder (ANB), (2) Alive Breeder (AB), (3) Dead as Non-Breeder (DNB), (4) Dead as Breeder (DB), and 5) the Absorbing State (AS). Note that there is one unobservable state in this definition: the Absorbing State (AS). The AS lacks a biological meaning but is used in multistate modelling to group all those individuals that enter a state that cannot be observed anymore throughout the course of the study. Transitions in the state matrix and relationships in the observation matrix were defined using parameters with different structures to match the biological and demographic features of the species (Fig. 7), which are described hereunder.

#### Survival, recruitment, and recovery

We modelled survival (*φ*) as time-constant but varying by sex and breeding status. Survival of breeders (*φ*_B_) was modelled as constant. For JOINT-ALL models, we modelled *φ*_B_ as varying between inside and outside the study area since we incorporated observations of breeders from the whole range of the species,. Survival of non-breeders (*φ*_NB_) was structured by age classes to accommodate typical age-related variation in long-lived populations and to be able to assess which population fractions were most affected by permanent emigration. Specifically, non-breeder survival was estimated separately for juveniles (1yo), immatures and subadults (2–3 yo) and adults (> = 4 yo), following Hernández-Matías et al.^52^. Sexual maturity was modelled as the probability of joining the breeding population, that is, occupying a breeding territory, so-called “recruitment” (γ). Recruitment was modelled as a sex and age-varying probability for juveniles (1yo), immatures (2yo), subadults (3yo) and adults (> = 4yo) (see^34^). The recovery parameter (r) was defined as the probability of encountering a dead marked individual. We modelled it as time-constant, but different for breeders (r_B_) and non-breeders (r_NB_) to accommodate any variation that may arise from differences in mortality causes associated with differences in behaviour and risk exposure. While recovery has traditionally been modelled in the observation matrix, we modelled it in the state transition matrix to avoid reported convergence issues in Bayesian hierarchical models^50^.

#### Recapture parameters

Recapture probabilities (i.e., probability of observing a live tagged individual) were modelled differently for non-breeders and breeders. Recapture of non-breeders (P_NB_) was modelled as constant across sex, time, and age classes. In addition, to accommodate the effects of breeder detection heterogeneity, we divided recapture probabilities of breeders into two parameters: 1) P_B1_: the probability of observing a ringed breeder for the first time, and 2) P_B_: the probability of observing it in subsequent occasions once it was observed for the first time^50^. Since breeders are only monitored during the breeding season, which occurs entirely during the first half of the year (i.e., January to June), P_B_ and P_B1_ were set as 0 at the second halves of the years (i.e., July to December). P_B1_ varied by territory of recruitment of each individual and time (see details about its estimation below in this section), and P_B_ varied by year using random effects to accommodate variations in territory sampling effort. In JOINT-ALL models, since we incorporated observations of all the distribution range, we differentiated P_B_ between Catalonia (yearly variation, random effects) and outside (constant probability).

The division of breeder recapture probabilities between P_B_ and P_B1_ was made because, given the system of monitoring implemented in the study area, monitoring teams would put more efforts into recontacting tagged breeders that had been observed previously in a certain territory. This fact would generally make newly recruited tagged birds less likely to be observed for the first time. This detection heterogeneity was represented using a parameterisation into the observation matrix that incorporated an individual and time-varying binary covariate $covp{b}_{i,t}$ along with P_B_ and P_B1_ to model the breeder observation process (Fig. 7; Figures S1, S2).

$$P\left(Observed\,as\,AB \right| State\,A{B)}_{i,t}=\left({P}_{B{1}_{ter{r}_{i},t}}*\left(1-covp{b}_{i,t}\right)\right)+({P}_{{B}_{t} }*covp{b}_{i,t})$$

For every individual, $covp{b}_{i,t}$ would take value 0 before and at the occasion of first detection of a breeder, and 1 at subsequent occasions. Hence, when $covp{b}_{i,t}$= 0, first-time breeder recapture probability P_B1_ was used to model the observation process conditional on the individual having recruited as a breeder but not yet observed, or observed for the first time. Instead, when $covp{b}_{i,t}$= 1 (i.e., all along after first breeder detection), P_B_ was applied. In cases where an individual was never observed as a live breeder, $covpb$ consisted of a row of zeros.

Importantly, P_B1_ may be poorly estimable from CMR data alone, as it refers to a single observation phenomenon (i.e., the first observation of a recruit), and thus to estimate it we used additional data. In our study system, each territory was routinely monitored every breeding season using either (1) telescope watching routines, (2) camera-trapping, or (3) a combination of both methods. All three methods followed standardized protocols in terms of the number of monitoring days per territory and breeding season, so that the observation effort was similar across territories and breeding seasons for each method. The chances of detecting a ringed breeder for the first time basically depend on the effectiveness at detecting ringed birds of the specific monitoring routine used in a territory during the breeding season. During monitoring routines, birds are searched inside territories by either of the three methods to try to ascertain whether they wear alphanumeric rings. If they do, additional efforts are carried out to read the alphanumeric codes in order to individually identify every breeder. Hence, to estimate P_B1_, we modelled information written down during the 2008–2020 monitoring surveys about the success/failure at distinguishing with certainty whether or not a breeding individual wore an alphanumeric ring in their tarsus when a territory surveillance season was performed with either telescopes, camera-traps, or both methods altogether. We assumed that the probability to first detect a ringed breeder was equal to the probability of distinguishing with certainty if it wore an alphanumeric ring, as in all cases rings were read after noticing that they were present in new breeders. We analysed these data with a logistic regression using ascertained ring presence/absence in an individual during a breeding season (*plr*_*i*_*)* as a response variable indicating (0): ring presence/absence could not be ascertained, and (1): ring presence/absence was ascertained. We used the monitoring methods as explanatory variables (*obs* for territory monitored using telescope routines and *camtrap* for camera trapping monitoring routines) indicating (1): method used, and (0): method not used.

$$logit\left(pl{r}_{i}\right)= \alpha + {\beta }_{1}*ob{{s}_{terr}}_{i}+{\beta }_{2}* camtra{{p}_{terr}}_{i}$$

The resulting estimates of effectiveness of each method were assigned to each territory depending on the type of monitoring used at each. Hence, P_B1_ would take a different value for each breeder depending on their territory of recruitment. In total, 55 (72%) territories were monitored with telescope routines only, 7 (9%) territories with camera trapping only, and 14 (19%) territories with both methods.

In SEP-CAT and JOINT-CAT models, the types of monitoring used in all territories were known with certainty. Since we aimed to reproduce a scenario without monitoring information outside our focal area, all territories outside Catalonia were assigned as unmonitored. Instead, when fitting the JOINT-ALL models, we did not have complete information about which specific territories were unmonitored or monitored, or by which method, in order to implement P_B1_. Therefore, we made reasonable modelling assumptions to reproduce the real conditions of monitoring. First, we assigned as monitored by any method or unmonitored those territories we had information about (n = 106, 30 of which outside the study area). In addition, we obtained information about the percentage of monitored territories and the frequency of usage of each method in each monitored population in Western Europe from Hernández-Matías et al.^3^ and updated unpublished information. We dealt with this uncertainty by modelling the monitoring status of each territory by each method as a Bernoulli-distributed variable with a probability equal to the percentage of monitored territories per region:

$$ob{s}_{terr} \sim Bernoulli(\%obs\left[regio{n}_{terr}\right])$$

$$camtra{p}_{terr} \sim Bernoulli(\%camtrap\left[regio{n}_{terr}\right])$$

where *region* stands for each of the different regions with Bonelli’s eagles present in western Europe and *terr* stands for each territory included in the models.

### The spatial submodel

#### Territories and natal dispersal

The distribution of breeding territories in Western Europe was represented as a point process, where each point corresponds to the location of a Bonelli’s eagle nest occupied during this study. Note that due to the strong fidelity of the study species to territories and nesting sites, territory and even nest locations rarely vary from year to year, which allow the definition of a territory as a static point^52^. Exact coordinates of breeding territories were available for all territories in the study population (i.e., Catalonia, n = 76) as well as for populations in Portugal (n = 93) and France (n = 44). For the rest of populations, all of them in Spain, territory locations were not known with certainty, and thus were simulated using updated data about the distribution and number of territories in different regions^51^. First, we used 10 × 10 km presence/absence grid data for the species in Spain^51^ and smoothed the data into a distribution map assuming territories would be circular with a 7-km radius (value obtained from radiotracking data). Then, following Atlas data on the number of territories per Spanish province for the same period^51^, we simulated territory locations inside the distribution areas of the species in every province, assuming a minimum distance between territories of 2 km. In total, 702 territory locations were simulated, which adds up to a total of 916 territories considered in this study (Figure S3).

Natal dispersal was measured as the geodesic distance between the territories of birth and recruitment. The territories of birth of all individuals were known with certainty as all birds were ringed as chicks. We modelled natal dispersal as different by sex, since raw data suggested that females tended to disperse further away^31,34^. Dispersal was modelled under the assumption of isotropical conditions (i.e., dispersal direction was uniform along the distribution range of the species), and therefore dispersal distributions needed to be strictly positive. The isotropical modelling was chosen due to the large numbers of territories available along the distance in the western European range of the species and the observed large dispersal capacity of the species in relation to the distance between birth and potential recruitment territories (Figure S4), which may help avoiding inconsistencies when using this modelling approach. We chose both a gamma and a lognormal distribution as dispersal kernel candidate functions. The gamma distribution was chosen due to its flexibility in modelling from exponential dispersal patterns to right-skewed kernels showing relatively high dispersal probabilities at large distances. The lognormal distribution was selected to represent right-skewed, fat-tailed dispersal kernels with higher dispersal probabilities at large distances. Both distributions were truncated at 1200 km because (1) no birds have ever been observed beyond that distance from a birth site (own data), and (2) this goes slightly far beyond the maximum distance between birth territories in the study area and other territories in SW Europe (ca. 1180 km). As for dispersal priors, we chose weakly informative priors that provided practically identical prior distance expectations for both distributions. We based our prior choices on published literature on natal dispersal in this species^34^. We performed prior predictive checks to ensure priors on distribution parameters were providing very similar and realistic prior dispersal distances^57,58^. In addition, we compared our final prior choices to alternative prior sets, and we checked that the sensitivity of our models’ posterior inferences to different prior choices was robust^57,58^. Our prior selection and analysis workflow for dispersal parameters is described in detail at Appendix S4 and Figures S12–S16 therein. To represent territorial recruitment, we provided *DM*: a 51 × 916 matrix with geodesic distances between all territories where chicks had ever been ringed during the study period (n = 51) and potential territories of recruitment (n = 916: 76 in Catalonia, 840 outside). We modelled natal dispersal distances for every individual as random draws from the sex-specific dispersal kernels. Next, we compared every individual’s drawn distance to the whole set of distances between the individual’s territory of birth and potential territories of recruitment provided in *DM*, and we assigned each recruit to the territory that showed the closest value to the drawn distance.

### Joint estimation of natal dispersal kernel and detection probabilities

The complexity of the permanent emigration challenge stems from the interplay between detection and dispersal probabilities^23,25^. Essentially, the observed natal dispersal kernel from a marked population in a study area restricted in size (*DK*_*o*_) is the result of the interaction between the true natal dispersal kernel (*DK*_*T*_) and the probability that natal dispersal events end up inside the study area (*W*) conditional on the location of departure of every dispersal movement (territory of birth in this case) and dispersal distance: $D{K}_{o}=D{K}_{t}*W$. The smaller the study area is, the greater the probability that a dispersal event will end up outside the boundaries of a study area and thus be indetectable, which may increase the difference between *DK*_*o*_ and *DK*_*t*_. Hence, the joint estimation formulation focuses on addressing this interplay. To do so, we first inform the model about which territories are inside or outside the study area. We do this with the binary vector *rp*, which indicates whether each of all 916 territories modelled in our study is (1) monitored (i.e., inside the study area), or (0) unmonitored (i.e., outside). We simulate natal dispersal distances for every individual as random draws from the kernel distribution and assign each breeder to a territory using the distance information provided by matrix *DM*. Next, we generate the binary vector P_d_, which using the information provided by *rp*, stores whether the territory of recruitment of each individual is (1) monitored/inside the study area, or (0) unmonitored/outside. Hence, P_d_ contains the information about every individual’s likelihood to remain or leave the study area (*W*), which is the key to estimate the true dispersal kernel DK_T_. Importantly, since P_d_ is individual-specific, it accounts for the fact that individuals will be more or less likely to remain in the study area after natal dispersal depending on their area of birth (i.e., the border effect). To act as a link between the spatial and the CMR submodels, P_d_ is incorporated into the state transition matrices in JOINT models (Figs. 6, S1). There, P_d_ is included to the transition from *t* to *t* + *1* for each individual *i* from state *Alive Non Breeder* to either *Alive* *Breeder* (i.e., breeder and inside the study area, thus detectable) if P_d_ = 1, or the *Absorbing State* (i.e., breeder and outside the study area, thus permanently undetectable) if P_d_ = 0, conditional on survival and recruitment. The Absorbing State (AS) retains all those individuals that enter a permanently unobservable state (see in the state matrix, Appendix S1, that the probability of transitioning from AS at *t* to AS at *t* + *1* equals 1). Traditionally, the AS has been used to indicate that dead individuals will remain dead and unobservable forever. Modelling-wise, the permanent emigration process is equivalent to mortality, as individuals that have left the study area will remain unobservable forever^32^. Hence, by modelling permanent emigration as a transition to the AS, whenever a non-breeder is not observed anymore throughout the course of the study, the model estimates the chances of it (1) being alive in the study area while remaining undetected, (2) having permanently emigrated from the study area, conditional to its natal dispersal distance, and (3) being dead and unrecovered. This approach allows the model to estimate the true dispersal kernel while dealing with the uncertainty present in the system (Fig. 6).

### Mapping permanent emigration by sex, territory, and distribution area

Based on the dispersal kernels by sex provided by the models, we performed simulations to estimate and map the continuous probability of permanent emigration by territory and sex. Simulations allowed us to make both males and females be born homogeneously across all territories in the study area (n = 76). To model natal dispersal, we used the sex-specific lognormal dispersal kernel estimates and their associated uncertainties obtained at JOINT-ALL. We built a model simulating natal dispersal events from males and females from each territory following the same st ructure as in the rest of models: natal dispersal distances were simulated as random draws from kernel distributions, recruitment was simulated following the distance information from matrix *DM*, and we again made use of vector *rp* to indicate whether each territory of recruitment was either inside or outside the study area. Territory-specific permanent emigration probabilities were calculated as the probabilities of recruiting outside the study area from each birth territory. Next, we mapped these space-discrete (i.e., territory-specific) probabilities and used a Gaussian process regression (spatial kriging) for interpolation^59^ to represent permanent emigration as a continuous probability along the distribution area of the study population. Interpolation was performed with ArcGIS Pro 2.5.2^60^.

### Model implementation

We checked for any deviations from the main assumptions of multistate models using Jolly-Movement (JMV) goodness-of-fit tests in software U-CARE 2.3.4^61^. As for Bayesian models, Gamma models (i.e., SEP-CAT-Gamma, JOINT-CAT-Gamma and JOINT-ALL-Gamma) were run for 4 chains of 70,000 iterations each, of which the first 50,000 were discarded. Lognormal models were run for 4 chains of 120,000 iterations each, and we discarded the first 100,000. Simulations to map permanent emigration probabilities were run for 4 chains of 100,000 iterations, of which the first 80,000 were discarded. All models were run in NIMBLE 0.12.1^54^ and R 4.1.2. Results are presented as medians followed by 89% Highest Posterior Density Intervals (HPDI^56^) in brackets.

## Supplementary Information

Supplementary Information.

## Data Availability

The code used during the current study is available in the figshare repository, https://figshare.com/s/ec4e606c21f774c8cf43. The datasets analysed during the current study are available in the same figshare repository upon publication date.

## References

[1] Sæther BE, Bakke O (2000). Avian life history variation and contribution of demographic traits to the population growth rate. Ecology.

[2] Matthysen, E. Multicausality of dispersal: A review. In *Dispersal Ecology and Evolution* (ed. Clobert, J.) 3–18 (Oxford University Press, Oxford, 2012).

[3] Hernández-Matías A (2013). From local monitoring to a broadscale viability assessment: A case study for the Bonelli’s Eagle in western Europe. Ecol. Monogr..

[4] Kubisch A, Holt RD, Poethke H-J, Fronhofer EA (2014). Where am I and why? Synthesizing range biology and the ecoevolutionary dynamics of dispersal. Oikos.

[5] McCaslin HM, Caughlin TT, Heath JA (2020). Long-distance natal dispersal is relatively frequent and correlated with environmental factors in a widespread raptor. J. Anim. Ecol..

[6] Paquet M, Arlt D, Knape J, Low M, Forslund P, Pärt T (2020). Why we should care about movements: Using spatially explicit integrated population models to assess habitat source–sink dynamics. J. Anim. Ecol..

[7] Morris, W. F. & Doak, D. F. in *Quantitative Conservation Biology: The Theory and Practice of Population Viability Analysis.* (Sinauer, Sunderland, Massachusetts, USA, 2002).

[8] Van Houtan KS, Bass OL, Lockwood J, Pimm SL (2010). Importance of estimating dispersal for endangered bird management. Conserv. Lett..

[9] Sutherland WJ, Pullin AS, Dolman PM, Knight TM (2004). The need for evidence-based conservation. Trends Ecol. Evol..

[10] Lebreton J-D, Burnham KP, Clobert J, Anderson DR (1992). Modeling survival and testing biological hypotheses using marked animals: A unified approach with case studies. Ecol. Monogr..

[11] Badia-Boher JA (2019). Evaluating European LIFE conservation projects: Improvements in survival of an endangered vulture. J. Appl. Ecol..

[12] Pradel R, Hines JE, Lebreton J-D, Nichols JD (1997). Capture-recapture survival models taking account of transients. Biometrics.

[13] O’Brien S, Robert B, Tiandry H (2005). Consequences of violating the recapture duration assumption of mark-recapture models: A test using simulated and empirical data from an endangered tortoise population. J. Appl. Ecol..

[14] Pradel R (2005). Multievent: An extension of the multistate capture-recapture models to uncertain states. Biometrics.

[15] Benton TG, Bowler DE, Clobert J (2012). Linking dispersal to spatial dynamics. Dispersal Ecology and Evolution.

[16] Gilroy JJ, Virzi T, Boulton RL, Lockwood JL (2012). A new approach to the “apparent survival” problem: Estimating true survival rates from mark–recapture studies. Ecology.

[17] Schaub M, Gimenez O, Schmidt BR, Pradel R (2004). Estimating survival and temporary emigration in the multistate capture-recapture framework. Ecology.

[18] Zimmerman GS, Gutiérrez RJ, Lahaye WS (2007). Finite study areas and vital rates: Sampling effects on estimates of spotted owl survival and population trends. J. Appl. Ecol..

[19] Marshall MR, Diefenbach DR, Wood LA, Cooper RJ (2004). Annual survival estimation of migratory songbirds confounded by incomplete breeding site–fidelity: Study designs that may help. Anim. Biodivers. Conserv..

[20] Barker RJ (1997). Joint modelling of live-recapture, tag-resight, and tag-recovery data. Biometrics.

[21] Lindberg MS, Kendall WL, Hines JE, Anderson MG (2001). Combining band recovery data and Pollock's robust design to model temporary and permanent emigration. Biometrics.

[22] Kendall WL (2013). Combining dead recovery, auxiliary observations and robust design data to estimate demographic parameters from marked individuals. Methods Ecol. Evol..

[23] Baker M, Nur N, Geupel GR (1995). Correcting biased estimates of dispersal and survival due to limited study area: Theory and an application using Wrentits. The Condor.

[24] Terui A (2020). Modeling dispersal using capture–recapture data: A comparison of dispersal models. Ecol. Res..

[25] Chadœuf J (2018). Modelling unbiased dispersal kernels over continuous space by accounting for spatial heterogeneity in marking and observation efforts. Methods Ecol. Evol..

[26] Schaub M, Royle JA (2014). Estimating true instead of apparent survival using spatial Cormack–Jolly–Seber models. Methods Ecol. Evol..

[27] Calvert AM (2009). A hierarchical Bayesian approach to multi-state mark-recapture: Simulations and applications. J. Appl. Ecol..

[28] Royle JA, Chandler RB, Sollmann R, Gardner B (2014). Spatial Capture-Recapture.

[29] Van Houtan KS, Pimm SL, Halley JM, Bierregaard RO, Lovejoy TE (2007). Dispersal of Amazonian birds in continuous and fragmented forest. Ecol. Lett..

[30] Nathan R, Klein E, Robledo-Arnuncio JJ, Revilla E, Clobert J (2012). Dispersal kernels: Review. Dispersal Ecology and Evolution.

[31] Clarke AL, Saether BE, Roskaft E (1997). Sex biases in avian dispersal: a reappraisal. Oikos.

[32] Dupont P, Allainé D, Ferrandiz-Rovira M, Pradel R (2022). Efficient spatial multi-state capture-recapture model to study natal dispersal: An application to the Alpine Marmot. J. Anim. Ecol..

[33] Real J, Mañosa S (2001). Dispersal of juvenile and immature Bonelli’s eagles in northeastern Spain. J. Raptor Res..

[34] Hernández-Matías A (2010). Determinants of territorial recruitment in Bonelli’s Eagle (Aquila fasciata) populations. Auk.

[35] Gelman A, Hill J (2007). Data Analysis using Regression and Multilevel/Hierarchical Models.

[36] Melbourne BA, Hastings A (2008). Extinction risk depends strongly on factors contributing to stochasticity. Nature.

[37] Penteriani V, Ferrer M, Delgado MM (2011). Floater strategies and dynamics in birds, and their importance in conservation biology: Towards an understanding of nonbreeders in avian populations. Anim. Conserv..

[38] Hernández-Matías A, Real J, Parés F, Pradel R (2015). Electrocution threatens the viability of populations of the endangered Bonelli’s eagle (Aquila fasciata) in Southern Europe. Biol. Cons..

[39] Trochet A, Courtois EA, Stevens VM, Baguette M (2016). Evolution of sex-biased dispersal. Q. Rev. Biol..

[40] Morandini V, Dietz S, Newton I, Ferrer M (2019). The role of age of first breeding in modeling raptor reintroductions. Ecol. Evol..

[41] Fandos G, Talluto M, Fiedler W, Robinson RA, Thorup K, Zurell D (2023). Standardised empirical dispersal kernels emphasise the pervasiveness of long-distance dispersal in European birds. J. Anim. Ecol..

[42] Zimmermann NE, Edwards TC, Graham CH, Pearman PB, Svenning JC (2010). New trends in species distribution modelling. Ecography.

[43] Ergon T, Gardner B (2014). Separating mortality and emigration: modelling space use, dispersal and survival with robust-design spatial capture-recapture data. Methods Ecol. Evol..

[44] McCarthy MA, Possingham HP (2007). Active adaptive management for conservation. Conserv. Biol..

[45] Greenwood PJ (1980). Mating systems, philopatry and dispersal in birds and mammals. Anim. Behav..

[46] Paradis E, Baillie SR, Sutherland WJ, Gregory RD (1998). Patterns of natal and breeding dispersal in birds. J. Anim. Ecol..

[47] McCarthy MA, Masters P (2005). Profiting from prior information in Bayesian analyses of ecological data. J. Appl. Ecol..

[48] Hanski I, Mononen T (2011). Eco-evolutionary dynamics of dispersal in spatially heterogeneous environments. Ecol. Lett..

[49] Azpillaga M, Real J, Hernández-Matías A (2018). Effects of rearing conditions on natal dispersal processes in a long-lived predator bird. Ecol. Evol..

[50] Kéry M, Schaub M (2012). Bayesian Population Analysis Using WinBUGS: A Hierarchical Perspective.

[51] Del Moral, J.C. & Molina, B. in *El águila perdicera en España, población reproductora en 2018 y método de censo*. (SEO/BirdLife, Madrid, Spain, 2018).

[52] Hernández-Matías A, Real J, Pradel R, Ravayrol A, Vincent-Martin N (2011). Effects of age, territoriality and breeding on survival of Bonelli’s Eagle Aquila fasciata. Ibis.

[53] Redondo-Gómez D (2022). Towards accurate and simple morphometric sex differentiation in Bonelli’s eagle Aquila fasciata nestlings: Interpopulation variations and influence of growth conditions. Avian Biol. Res..

[54] de Valpine P, Turek D, Paciorek CJ, Lang DT, Bodik R (2016). Programming with models: Writing statistical algorithms for general model structures with NIMBLE. J. Comput. Graph. Stat..

[55] Ver Hoef JM, Boveng PL (2015). Iterating on a single model is a viable alternative to multimodel inference. J. Wildl. Manag..

[56] McElreath, R. In *Statistical Rethinking: A Bayesian Course with Examples in R and STAN.* (CRC Press, Boca Raton, FL, 2019).

[57] Lemoine NP (2019). Moving beyond noninformative priors: why and how to choose weakly informative priors in Bayesian analyses. Oikos.

[58] Wesner JS, Pomeranz JPF (2021). Choosing priors in Bayesian ecological models by simulating from the prior predictive distribution. Ecosphere.

[59] Schulz E, Speekenbrink M, Krause A (2018). A tutorial on Gaussian process regression. J. Math. Psychol..

[60] ESRI. ArcGIS Pro: Release 2.5.2. (Environmental Systems Research Institute, Redlands, CA, 2020).

[61] Choquet R, Lebreton JD, Gimenez O, Reboulet AM, Pradel R (2009). U-CARE: Utilities for performing goodness of fit tests and manipulating capture-recapture data. Ecography.

