# Supplementary material for: Joint estimation of survival and dispersal effectively corrects the permanent emigration bias in mark-recapture analyses
Source: Sci Rep. 2023 Apr 28;13:6970. doi: 10.1038/s41598-023-32866-0 (PMC10147689; doi:10.1038/s41598-023-32866-0)
Supplement: Supplementary file 1 — Supplementary Information. [file 41598_2023_32866_MOESM1_ESM.pdf]

Joint estimation of survival and dispersal effectively corrects the permanent emigration bias in mark-recapture analyses

Badia-Boher, J.A., Real, J., Riera, J.L., Bartumeus F., Parés, F., Bas, J.M., & Hernández-Matías, A.

Scientific Reports

Supplementary Material S1: Multistate submodel - state and observation matrices

Figure S1. State transition matrix of the multistate joint-estimation models. Rows indicate state of individual i at time t, columns indicate state of individual i at time t+1. The matrix includes the parameters φ_{NB} (non-breeder survival), φ_B (breeder survival), γ (recruitment to the breeding population), P_d (joint-estimation parameter), and r (probability of encountering a dead marked individual that was either non-breeder, r_{NB}, or breeder, r_B). Subindices indicate age ($_a$), sex ($_s$), individual ($_i$), and time ($_t$). States are Alive as Non-Breeder (ANB), Alive as Breeder (AB), Dead as Non-Breeder (DNB), Dead as Breeder (DB), and Absorbing State (AS). Due to the length of the first absorbing state expression (i.e., transition from ANB in t to AS in t+1), we wrote this expression outside the matrix ($*_A$). Note that r (recovery) is indeed an observation parameter and is often modelled in the observation matrix, but for convergence issues in Bayesian models it must be modelled in the state matrix. Refer to Kéry and Schaub (2012) for further information.

t / t+1	ANB	AB	DNB	DB	AS
ANB	$(\phi_{NB_{a,s}} * (1-\gamma_{a,s}))$	$(\phi_{NB_{a,s}} * \gamma_{a,s} * P_{d_i})$	$((1-\phi_{NB_{a,s}}) * (1-\gamma_{a,s}) * r_{NB})$	$((1-\phi_{NB_{a,s}}) * (\gamma_{a,s}) * r_B)$	$*_A$
AB	0	ϕ_{B_s}	0	$(1-\phi_{B_s}) * r_B$	$(1-\phi_{B_s}) * (1-r_B)$
DNB	0	0	0	0	1
DB	0	0	0	0	1
AS	0	0	0	0	1

$$*A: ((1-\phi_{NB_{a,s}}) * (1-\gamma_{a,s}) * (1-r_{NB})) + (\phi_{NB_{a,s}} * \gamma_{a,s} * (1-P_{d_i})) + ((1-\phi_{NB_{a,s}}) * \gamma_{a,s} * (1-P_{d_i})) + ((1-\phi_{NB_{a,s}}) * \gamma_{a,s} * (1-r_B) * P_{d_i})$$

Figure S2. Observation matrix of the multistate models. Rows indicate state of individual i at time t, columns indicate observation/event of individual i at time t. The matrix includes the parameters P_{NB} (non-breeder recapture), P_{B1} (probability of being observed for the first time once the individual has become breeder), and P_B (breeder recapture for those individuals observed as breeders at least once already), and the binary covariate *covpb*, which assigns either P_{B1} or P_B as observation parameters to deal with detection heterogeneity in breeder detection probabilities. States are Alive as Non-Breeder (ANB), Alive as Breeder, Dead as Non-Breeder (DNB), Dead as Breeder (DB), and Absorbing State (AS). Observations/Events are Alive as Non-Breeder (1), Alive as Breeder (2), Recovered as Non-Breeder (3), Recovered as Breeder (4) and Unobserved/Unrecovered (5). Note that due to issues in model convergence in Bayesian hierarchical capture-mark-recapture models, we had to parameterize recovery probabilities in the state transition matrix. For further information on recovery parameterisations, check Kéry and Schaub (2012).

$$\begin{array}{c|ccccc} t/t & \mathbf{1} & \mathbf{2} & \mathbf{3} & \mathbf{4} & \mathbf{5} \\ \mathbf{ANB} & P_{NB} & 0 & 0 & 0 & 1-P_{NB} \\ \mathbf{AB} & 0 & P_{B1_{terr_i,t}} * (1-covpb_{i,t}) + P_{B_t} * covpb_{i,t} & 0 & 0 & 1-P_B \\ \mathbf{DNB} & 0 & 0 & 1 & 0 & 0 \\ \mathbf{DB} & 0 & 0 & 0 & 1 & 0 \\ \mathbf{AS} & 0 & 0 & 0 & 0 & 1 \end{array}$$

Joint estimation of survival and dispersal effectively corrects the permanent emigration bias in mark-recapture analyses

Badia-Boher, J.A., Real, J., Riera, J.L., Bartumeus, F., Parés, F., Bas, J.M., & Hernández-Matías, A.

Scientific Reports

Supplementary Material S2: Supplementary figures and tables of Methods and Results

Table S1. Main survival and average dispersal distance results by model.

Estimate	Age	Sex	Distribution	Model estimates: Median (89%HPDI)		
				**Sep-Cat**	**Joint-Cat**	**Joint-All**
**Avg. Disp. Distance (km)**	(all)	Female	Gamma	95.2 (65.3 – 123.6)	114.7 (72.4 – 155.8)	159.6 (121.2 – 194.7)
			Lognormal	124.7 (77.1 – 173.0)	221.0 (92.1 – 336.2)	213.6 (126.6 - 289.6)
	(all)	Male	Gamma	35.6 (27.9 - 43.1)	37.0 (28.8 – 45.9)	71.1 (51.1 – 89.9)
			Lognormal	46.5 (32.6 - 59.1)	52.8 (35.0 – 75.5)	73.3 (47.1 – 97.2)
**Non-Breeding Survival**	1 yo.	Female	Gamma	0.626 (0.529 - 0.725)	0.645 (0.546 - 0.747)	0.677 (0.588 - 0.770)
			Lognormal	0.627 (0.533 - 0.731)	0.667 (0.568 - 0.768)	0.674 (0.580 - 0.764)
	2-3 yo.	Female	Gamma	0.622 (0.522 - 0.720)	0.658 (0.553 - 0.761)	0.706 (0.618 - 0.788)
			Lognormal	0.626 (0.524 - 0.722)	0.684 (0.578 – 0.782)	0.701 (0.613 - 0.785)
	Adults	Female	Gamma	0.820 (0.645 - 0.979)	0.846 (0.681 - 0.992)	0.897 (0.781 - 0.990)
			Lognormal	0.818 (0.642 - 0.976)	0.857 (0.695 - 0.996)	0.892 (0.775 - 0.991)
	1 yo.	Male	Gamma	0.611 (0.509 - 0.711)	0.622 (0.523 - 0.723)	0.648 (0.555 - 0.742)
			Lognormal	0.621 (0.521 - 0.723)	0.641 (0.543 - 0.743)	0.644 (0.551 - 0.745)
	2-3 yo.	Male	Gamma	0.736 (0.644 - 0.828)	0.743 (0.652 - 0.832)	0.760 (0.671 - 0.844)
			Lognormal	0.739 (0.645 - 0.828)	0.759 (0.671 - 0.848)	0.758 (0.666 - 0.841)
	Adults	Male	Gamma	0.837 (0.717 - 0.947)	0.845 (0.721 - 0.948)	0.848 (0.735 - 0.952)
			Lognormal	0.839 (0.719 - 0.942)	0.846 (0.734 - 0.954)	0.848 (0.727 - 0.954)
**Territorial Survival**	(all)	Female	Gamma	0.879 (0.809 - 0.948)	0.874 (0.801 - 0.941)	0.870 (0.792 - 0.951)
			Lognormal	0.877 (0.805 - 0.944)	0.876 (0.801 - 0.942)	0.872 (0.800 - 0.940)
	(all)	Male	Gamma	0.886 (0.838 - 0.933)	0.887 (0.839 - 0.934)	0.886 (0.841 - 0.931)
			Lognormal	0.886 (0.834 - 0.932)	0.888 (0.838 - 0.934)	0.890 (0.845 - 0.937)

Table S2. Long-distance natal dispersal probability (i.e., distances higher than 200 km) by dispersal kernel and sex.

Sex	Distribution	Model estimates: Median (89%HPDI)		
		**Sep-Cat**	**Joint-Cat**	**Joint-All**
**Female**	Gamma	0.074<br>(0.006 – 0.165)	0.123<br>(0.017 – 0.271)	0.279<br>(0.178 – 0.385)
	Lognormal	0.143<br>(0.041 – 0.261)	0.288<br>(0.108 – 0.477)	0.306<br>(0.184 – 0.434)
**Male**	Gamma	0<br>(0 - 0)	0<br>(0 - 0)	0.054<br>(0.013 – 0.104)
	Lognormal	0.018<br>(0 – 0.045)	0.037<br>(0.002 – 0.102)	0.082<br>(0.028 – 0.151)

Table S3. Dispersal parameter estimates (i.e., shape and rate for gamma distributions, mean and standard deviation for lognormal distributions) by model type and sex.

Sex	Distribution	Parameter	Model estimates: Median (89%HPDI)		
			**Sep-Cat**	**Joint-Cat**	**Joint-All**
**Female**	Gamma	Shape	1.845 (1.205 – 2.547)	1.842 (1.166 – 2.534)	1.574 (1.123 – 2.035)
		Rate	0.020 (0.009 – 0.030)	0.017 (0.007 – 0.027)	0.010 (0.006 – 0.014)
	Lognormal	Mean	4.284 (3.951 – 4.644)	4.683 (4.213 – 5.249)	4.755 (4.412 – 5.098)
		sd	0.958 (0.729 – 1.203)	1.104 (0.804 – 1.421)	1.082 (0.859 – 1.332)
**Male**	Gamma	Shape	1.892 (1.325 – 2.509)	1.915 (1.321 – 2.543)	1.032 (0.752 – 1.311)
		Rate	0.054 (0.032 – 0.077)	0.052 (0.031 – 0.075)	0.015 (0.009 – 0.021)
	Lognormal	Mean	3.341 (3.084 – 3.605)	3.484 (3.164 – 3.848)	3.662 (3.366 – 3.971)
		sd	0.941 (0.758 – 1.134)	1.019 (0.778 – 1.269)	1.177 (0.965 – 1.424)

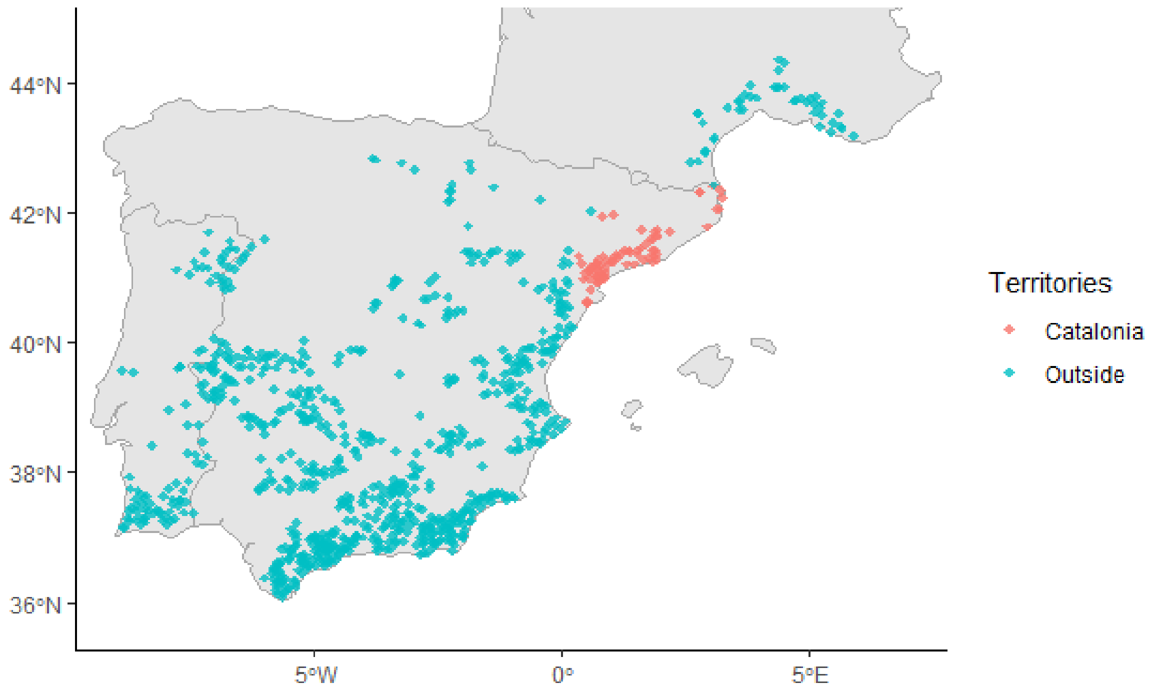

Figure S3. Locations of Bonelli's eagle territories in continental western Europe used in the spatial component of the models. Map source: Natural Earth.

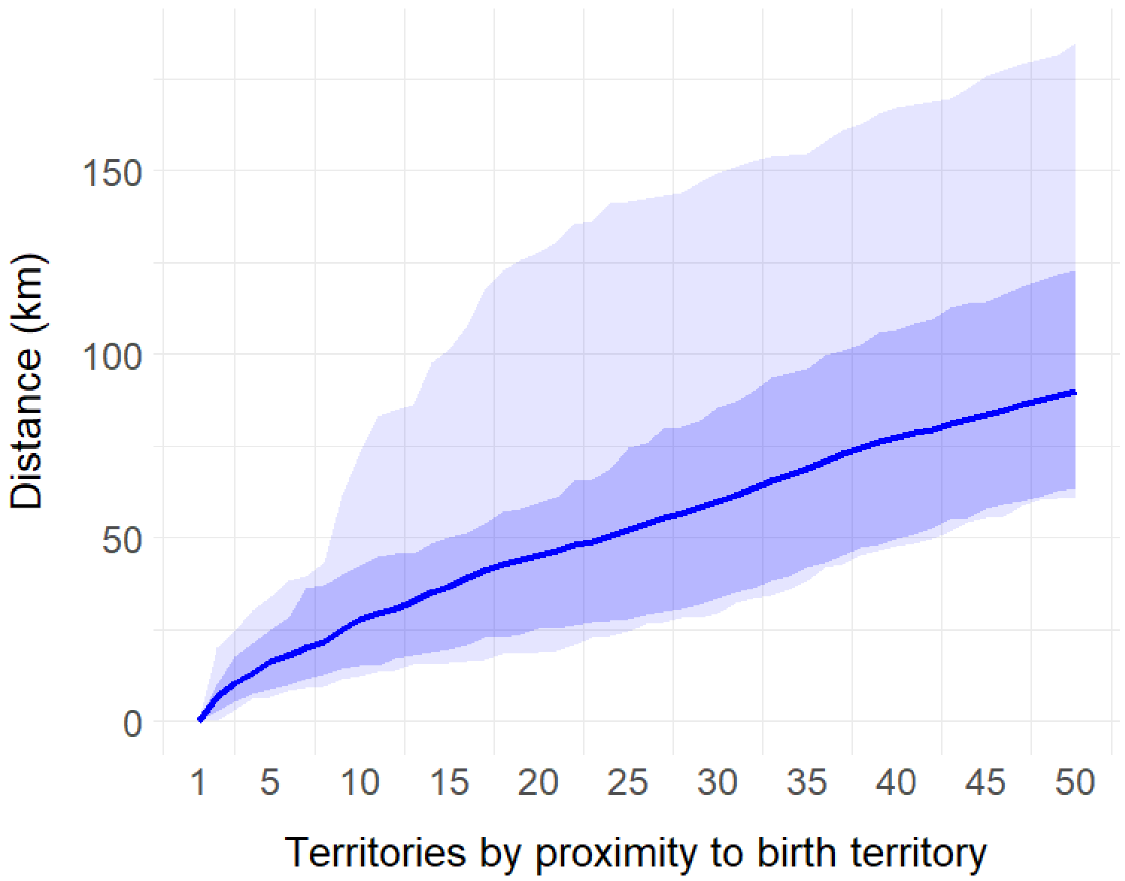

Figure S4. Mean (thick line), 85% quantile (strong blue shaded area), and 95% quantile (light blue shaded area) distance values between territories of birth and the 50 closest potential territories of recruitment.

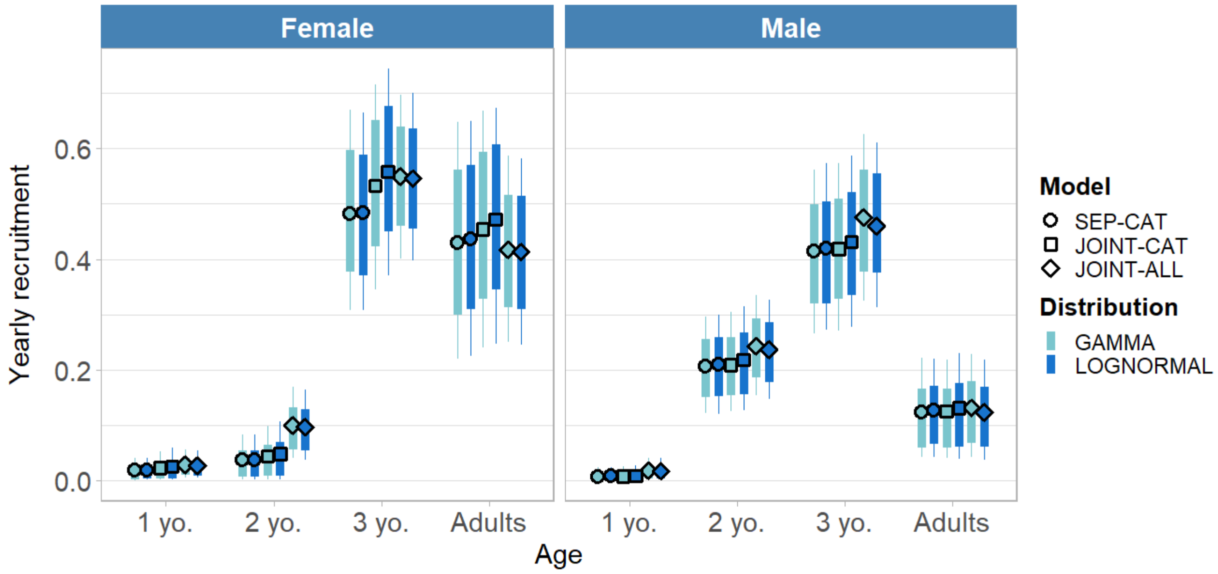

Figure S5. Recruitment probabilities by model, sex, and age. Circles and squares indicate median values. Thick lines indicate the 66% HPDI and fine lines show the 89% HPDI.

Joint estimation of survival and dispersal effectively corrects the permanent emigration bias in mark-recapture analyses

Badia-Boher, J.A., Real, J., Riera, J.L., Bartumeus, F., Parés, F., Bas, J.M., & Hernández-Matías, A.

Scientific Reports

Supplementary Material S3: A brief guide on adapting the Joint Estimation multistate matrices to different study systems

The joint estimation approach is a flexible formulation to address biases in demographic parameters caused by permanent emigration. This model formulation is divided into a spatial submodel and a multistate capture-mark-recapture (CMR) submodel. The spatial submodel models natal dispersal, while the CMR submodel provides information on demographic parameters such as survival and sexual maturity (i.e., age of first breeding or recruitment to the breeding population, among other possibilities). Both submodels are linked by a specific parameter that estimates individual probabilities of permanent emigration accounting for information provided by both submodels and detectability along space. This parameter is accommodated in the state matrices of the multistate submodel to represent the permanent emigration process. Whenever an individual achieves sexual maturity, it undertakes natal dispersal towards the area of breeding. If that site is outside the study area, this is considered as permanent emigration. Such process is modelled as a transition to the absorbing state (i.e., a state of permanent undetectability) as the individual at issue will not be observable anymore throughout the course of the study. Based on this modelling approach, to implement the joint estimation it is only necessary to model survival and sexual maturity in the state matrix of the multistate submodel (Figure S6). As for the observation matrix, a single recapture probability would be necessary, same for breeders and non-breeders (Figure S7). Note that except for the P_d parameter, which should be structured by individual in case we want to account for birth

territory locations and the border effect, structuring parameters by age, sex, or sexual maturity status as in our study, is not necessary either for implementing this method.

$$\begin{array}{cccc} t / t+1 & \textbf{ANB} & \textbf{AB} & \textbf{AS} \\ \textbf{ANB} & (\phi *\ (1-\gamma)) & (\phi *\ \gamma * P_{d_i}) & (1-\phi) + \left(\phi * \gamma * \left(1 - P_{d_i}\right)\right) \\ \textbf{AB} & 0 & \phi & 1-\phi \\ \textbf{AS} & 0 & 0 & 1 \end{array}$$

Figure S6. Simplest state matrix possible to implement the joint estimation approach. States ANB, AB and AS stand for Alive – Non Breeder, Alive – Breeder and Absorbing State respectively. Parameters are ϕ (survival), γ (sexual maturity), and the individual-specific permanent emigration parameter Pd_i.

$$\begin{array}{cccc} t / t+1 & \textbf{1} & \textbf{2} & \textbf{3} \\ \textbf{ANB} & P & 0 & 1-P \\ \textbf{AB} & 0 & P & 1-P \\ \textbf{AS} & 0 & 0 & 1 \end{array}$$

Figure S7. Simplest observation matrix possible to implement the joint estimation approach. States ANB, AB and AS stand for Alive – Non Breeder, Alive – Breeder and Absorbing State respectively, while observation codes 1, 2, 3 stand for Observed as Alive – Non Breeder, Observed as Alive – Breeder and Unobserved respectively. Parameter P stands for recapture probability.

The flexibility of the multistate capture-mark-recapture method may facilitate the adaptation of the joint estimation approach to other study designs. For instance, note that in Figure S6 we modelled the non-breeder to breeder transition as a stochastic probability, but if individuals become sexually mature at a fixed age (i.e., deterministically), this may also be adapted in the matrices using age states. As an example of an appropriate formulation, if an individual becomes sexually mature at the end of its second year of life, one may use the states "Alive – Non Breeder 1 year old", "Alive – Non Breeder 2 years old", "Alive – Breeder", and the Absorbing State (Figures S8 and S9). In this case, transitions from live states should only be

determined by survival, as the probability to transition from being non-breeder at year 1 to being non-breeder at year 2, and to being breeder at year 3, only depends on surviving from year to year.

t / t+1	ANB 1Y	ANB 2Y	AB	AS
ANB-1Y	0	ϕ	0	$1-\phi$
ANB-2Y	0	0	$\phi * P_{d_i}$	$(1-\phi)+\left(\phi * \left(1-P_{d_i}\right)\right)$
AB	0	0	ϕ	$1-\phi$
AS	0	0	0	1

Figure S8. State matrix adapting the multistate and joint estimation formulation to a species with a deterministic age of sexual maturity (at the end of the second year of life). States ANB – 1Y, ANB – 2Y, AB, and AS stand for Alive – Non Breeder at year 1, Alive – Non Breeder at year 2, Alive – Breeder, and Absorbing State respectively. Parameters are ϕ (survival), and the individual-specific permanent emigration parameter P_{d_i}.

t / t+1	1	2	3	4
ANB-1Y	P	0	0	$1-P$
ANB-2Y	0	P	0	$1-P$
AB	0	0	P	$1-P$
AS	0	0	0	1

Figure S9. Observation matrix adapting the multistate and joint estimation formulation to a species with a deterministic age of sexual maturity (at the end of the second year of life). States ANB – 1Y, ANB – 2Y, AB, and AS stand for Alive – Non Breeder at year 1, Alive – Non Breeder at year 2, Alive – Breeder, and Absorbing State respectively. Observation codes 1, 2, 3, and 4 stand for Observed at Alive – Non Breeder at year 1, Observed a Alive – Non Breeder at year 2, Observed as Alive – Breeder, and Unobserved respectively. Parameter P stands for recapture probability.

From these basic models, it is easy to add complexity to the state transition and observation matrices in further steps. For instance, from the simplest matrices presented here (Figures S6 and S7), we may add the modelling of recoveries of dead individuals (Figures S10 and S11. Recall that while recoveries have traditionally been modelled as observation parameters, in Bayesian hierarchical models and BUGS language we usually model them in the state matrices to avoid convergence issues.

$$
\begin{array}{c|cccc}
t\,/\,t+1 & \textbf{ANB} & \textbf{AB} & \textbf{D} & \textbf{AS} \\
\hline
\textbf{ANB} & (\phi*(1-\gamma)) & (\phi*\gamma*P_{d_i}) & (1-\phi)*r & (1-\phi)*(1-r)+\left(\phi*\gamma*\left(1-P_{d_i}\right)\right) \\
\textbf{AB} & 0 & \phi & (1-\phi)*r & (1-\phi)*(1-r) \\
\textbf{D} & 0 & 0 & 0 & 1 \\
\textbf{AS} & 0 & 0 & 0 & 1
\end{array}
$$

Figure S10. State matrix adapting the multistate and joint estimation formulation to a study design with recoveries of dead individuals. States ANB, AB, D, and AS stand for Alive – Non Breeder, Alive – Breeder, (Recently) Dead, and Absorbing State. Parameters are ϕ (survival), γ (sexual maturity), r (recovery), and the individual-specific permanent emigration parameter P_{d_i}.

$$
\begin{array}{c|cccc}
t\,/\,t+1 & \textbf{1} & \textbf{2} & \textbf{3} & \textbf{4} \\
\hline
\textbf{ANB} & P & 0 & 0 & 1-P \\
\textbf{AB} & 0 & P & 0 & 1-P \\
\textbf{D} & 0 & 0 & 1 & 0 \\
\textbf{AS} & 0 & 0 & 0 & 1
\end{array}
$$

Figure S11. Observation matrix adapting the multistate and joint estimation formulation to a study design with recoveries of dead individuals. States ANB, AB, D, and AS stand for Alive – Non Breeder, Alive – Breeder, (Recently) Dead, and Absorbing State respectively. Observation codes 1, 2, 3, and 4 stand for Observed as Alive – Non Breeder, Observed as Alive – Breeder, Observed as (Recently) Dead, and Unobserved respectively. Parameter P stands for recapture probability.

As shown, using the flexibility of multistate matrix we can add further layers of complexity to basic matrices in order to accommodate study systems with greater structural or modelling particularities. In the particular case of our study, we easily adapted the matrices to the particularities of the study species, with detection heterogeneity in breeding birds, and recovery probabilities. Further specificities of study populations may be added, such as disease status [1,2], body condition [3], and tag loss [4], among others.

References

[1] Senar, J.C., & Conroy, M.J. (2004). Multi-state análisis of the impacts of avian pox on a population of Serins (Serinus serinus): the importance of estimating recapture rates. *Animal Biodiversity and Conservation*, 27.1, 133-146.

[2] Conn, P.B., & Cooch, E.G. (2009). Multistate capture-recapture analysis under imperfect state observation: an application to disease models. *Journal of Applied Ecology*, 46, 486-492.

[3] Boulanger, J., Catter, M., Nielsen, S.E., Stenhouse, G., Cranston, J. (2013). Use of multistate models to explore relationships between changes in body condition, habitat and survival of grizzly bears Ursus arctos horribilis. *Wildlife Biology*, 19(3), 247-288.

[4] Badia-Boher, J.A., Sanz-Aguilar, A., de la Riva, M., Gangoso, L., van Overveld, T., García-Alfonso, M., Luzardo, O.P., Suarez-Pérez, A., & Donázar, J.A. (2019). Evaluating European LIFE conservation projects: Improvements in survival of an endangered vulture. *Journal of Applied Ecology*, 56, 1210–1219.

Joint estimation of survival and dispersal effectively corrects the permanent emigration bias in mark-recapture analyses

Badia-Boher, J.A., Real, J., Riera, J.L., Bartumeus, F., Parés, F., Bas, J.M., & Hernández-Matías, A.

Scientific Reports

Supplementary Material S4: Workflow, details, and analyses of our prior dispersal parameter choices

In this appendix, we discuss the prior choice for our dispersal distributions. First, we discuss our prior selection workflow. Second, we present our prior choices and discuss their adequacy above other alternative choices using data published in other studies and prior predictive checks. Third, we test the sensitivity of our models' posterior inference to prior choice.

The choice of prior distributions is an important step in Bayesian statistics and modelling [1,2,3]. In this study, we are comparing the results of models that rely on different dispersal kernel distributions. See the structure of our dispersal parameters in lognormal and gamma models:

$$Distance_{i,sex} \sim Lognormal(mean_{sex}, sd_{sex});$$

$$Distance_{i,sex} \sim Gamma(shape_{sex}, rate_{sex});$$

The prior distributions of the mean and standard deviation in the lognormal distribution and shape and rate in the gamma distribution will determine the shape of the prior dispersal kernels. Recall that in our study both distributions are truncated at a maximum value of 1200 km. A fundamental step is to find prior distributions that provide similar dispersal distance expectations for both models. This way, we can ensure priors do not affect the estimates of our models based on different distributions in significantly different ways. The information provided by prior beliefs in a model can be easily evaluated using prior predictive checks (PPC; [2,3]). PPCs are based on simulating and visualizing data from prior distributions. Using PPCs,

our aim was to choose weakly informative priors that 1) encompass the range of potential values of each parameter, 2) generate reasonable values of natal dispersal distances, that is, those that encompass the range of observed distances for the Bonelli's eagle in continental western Europe in published studies [4], and 3) regularize against dispersal distance values that are considered infrequent or have never been reported according to the literature [2,4,5].

Following this workflow, by educated trial-and-error following PPCs of a range of candidate values [3], we selected normally distributed priors for both lognormal and gamma parameters, all truncated at 0 since none of both distributions accept negative values.

$$mean_{sex} \sim Normal(\text{mean} = 2.3\,, \text{sd} = 1.5);\; sd_{sex} \sim Normal(\text{mean} = 0.6\,, \text{sd} = 0.7)$$

$$shape_{sex} \sim Normal(\text{mean} = 1.2, \text{sd} = 1.2);\; rate_{sex} \sim Normal(\text{mean} = 0.04, \text{sd} = 0.05)$$

The selected prior distributions result in almost identical prior natal dispersal kernels for both distributions (Figure S12). The standard deviations of our distribution parameters appear way larger than those of any potential posterior estimate with the amount of data available. In addition, the priors encompass the range of distances published in this and neighbouring populations and regularize against extremely large natal dispersal distances that have rarely been reported ([4]; Figure S13).

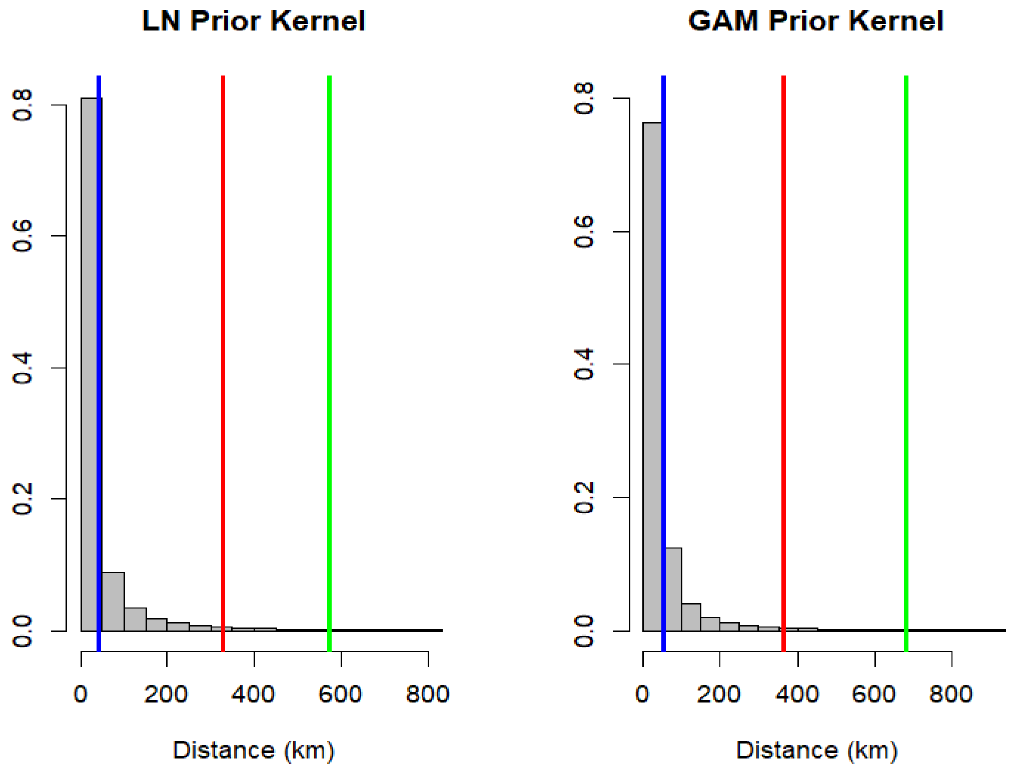

Figure S12. Mean (blue), quantile 97.5%, and quantile 99% values of lognormal (LN) and gamma (GAM) prior dispersal kernels using the selected priors.

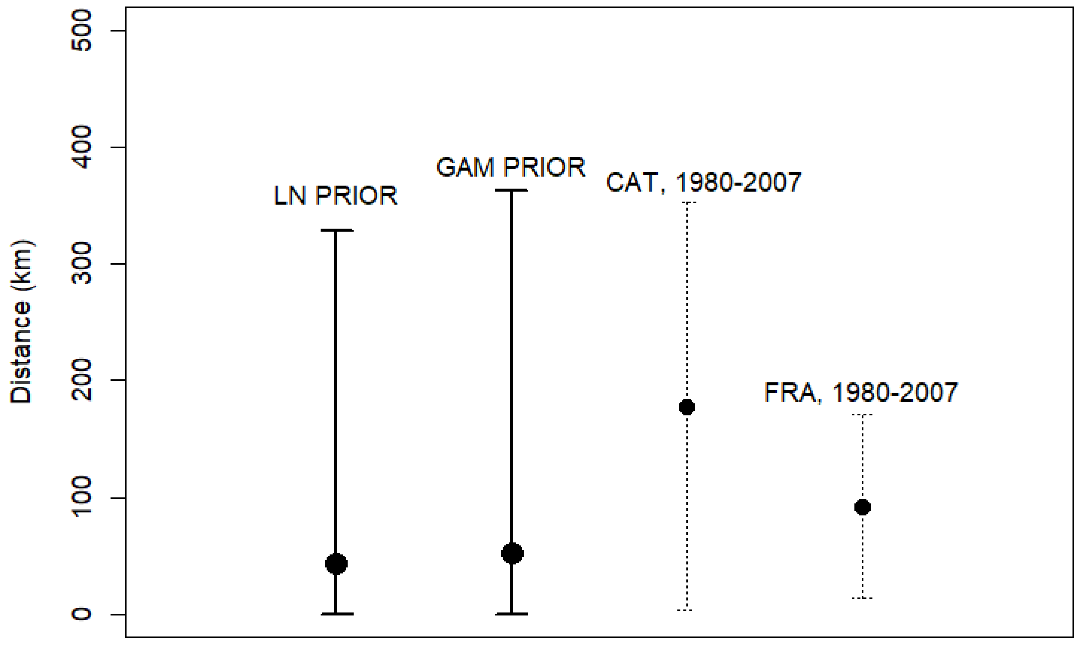

Figure S13. Mean and quantile 2.5% and 97.5% values of lognormal (LN) and gamma (GAM) prior dispersal kernels compared to the mean and equal quantiles of natal dispersal distances published for the study population, Catalonia (CAT) and the neighbouring population in France (FRA), Hernández-Matías et al. [4].

When compared to the published data in the study population and the neighbouring French population, the means of our prior dispersal kernels for both distributions are lower (Figure S13). This is because 1) dispersal distances on published articles were analysed using normal distributions, which have markedly different shapes to gamma and lognormal distributions, and 2) while published data do not account for sex, we will be splitting our dispersal estimations into sexes, and males presumably have shorter dispersal distances than females, thus we prefer to be conservative.

Although flat uniform priors may intuitively seem an adequate non-informative choice, in this situation they may even be strongly informative. Using PPCs, if we considered uniform prior distributions for both gamma and lognormal parameters that encompassed a range across and beyond the likely values of all parameters, say,

$$mean_{sex} \sim Uniform(\mathrm{min} = 0\,, \mathrm{max} = 10); sd_{sex} \sim Uniform(\mathrm{min} = 10\,, \mathrm{max} = 10)$$

$$shape_{sex} \sim Uniform(\mathrm{min} = 0, \mathrm{max} = 10);\ rate_{sex} \sim Uniform(\mathrm{min} = 0, \mathrm{max} = 10)$$

if we draw values from these prior distributions, considerable differences among distributions are generated: lognormal and gamma dispersal kernels had mean values of 4 vs 177 km, and 97.5% quantiles of 20 vs 1095 km respectively. Thus, the selected normally distributed priors are more robust choices in these cases.

One further possibility may be to use priors that reflect wider uncertainties about the dispersal kernels of both distributions between the range of distances considered in our study (i.e., 0-1200 km). Although this may also be an interesting choice, prior dispersal kernel distributions would then keep considerable prior probability densities at large and unlikely distances, which may not contribute to regularizing towards more likely values and thus not be as optimal as the priors selected in this study. However, an interesting exercise to understand the effect of our prior selection would be to analyse the sensitivity of prior choice on posterior inference (i.e.,

narrower weakly regularizing prior as the one chosen in our study and shown in Figure S12 vs. wider prior shown in Figure S14). We selected such wider priors by trial and error and using PPCs. We used normally-distributed priors truncated at 0 for each distribution, again focusing on obtaining similar prior distance expectations (lognormal and gamma respectively).

$$mean_{sex} \sim Normal(mean = 5.4, \mathrm{sd} = 1);\ sd_{sex} \sim Normal(\mathrm{mean} = 0.75\,, \mathrm{sd} = 0.3)$$

$$shape_{sex} \sim Normal(\mathrm{mean} = 2.9, \mathrm{sd} = 1);\ rate_{sex} \sim Normal(\mathrm{mean} = 0.01, \mathrm{sd} = 0.0115)$$

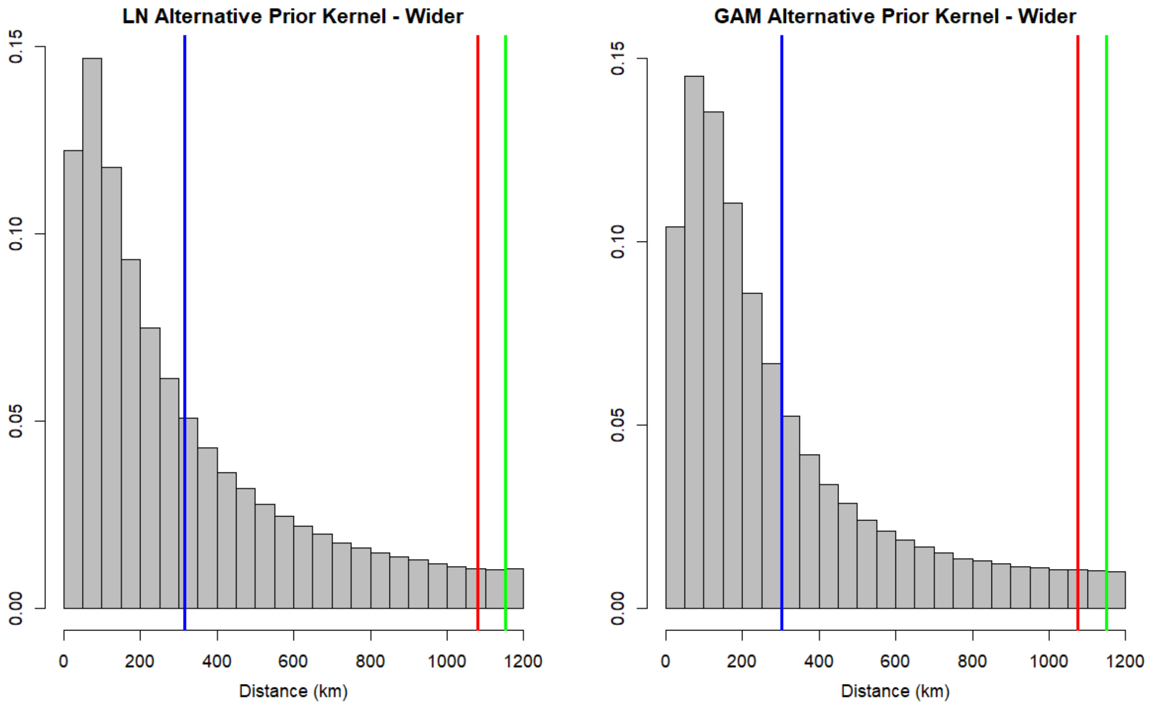

Figure S14. Mean (blue), quantile 97.5%, and quantile 99% values of lognormal (LN) and gamma (GAM) prior dispersal kernels using alternative wider priors.

These alternative dispersal kernels show median distances of 212 and 202 km, 2.5% quantile distances of 17 and 16 km, and 97.5% quantile distances of 1080 and 1076 km for the lognormal and the gamma distribution respectively – that is, both kernel distributions are very similar, and show way larger expectations than the ones selected in this study (Figure S14).

We compared posterior estimates of our models when using both sets of priors [1]. We did so for SEP-CAT and JOINT-CAT models (we excluded JOINT-ALL models because of their longer computation times). We compared the survival and average dispersal distance estimates obtained using both priors in the range of different models considered in this sensitivity analysis. We define the prior selected in our study as "Narrower" and the alternative prior described above as "Wider".

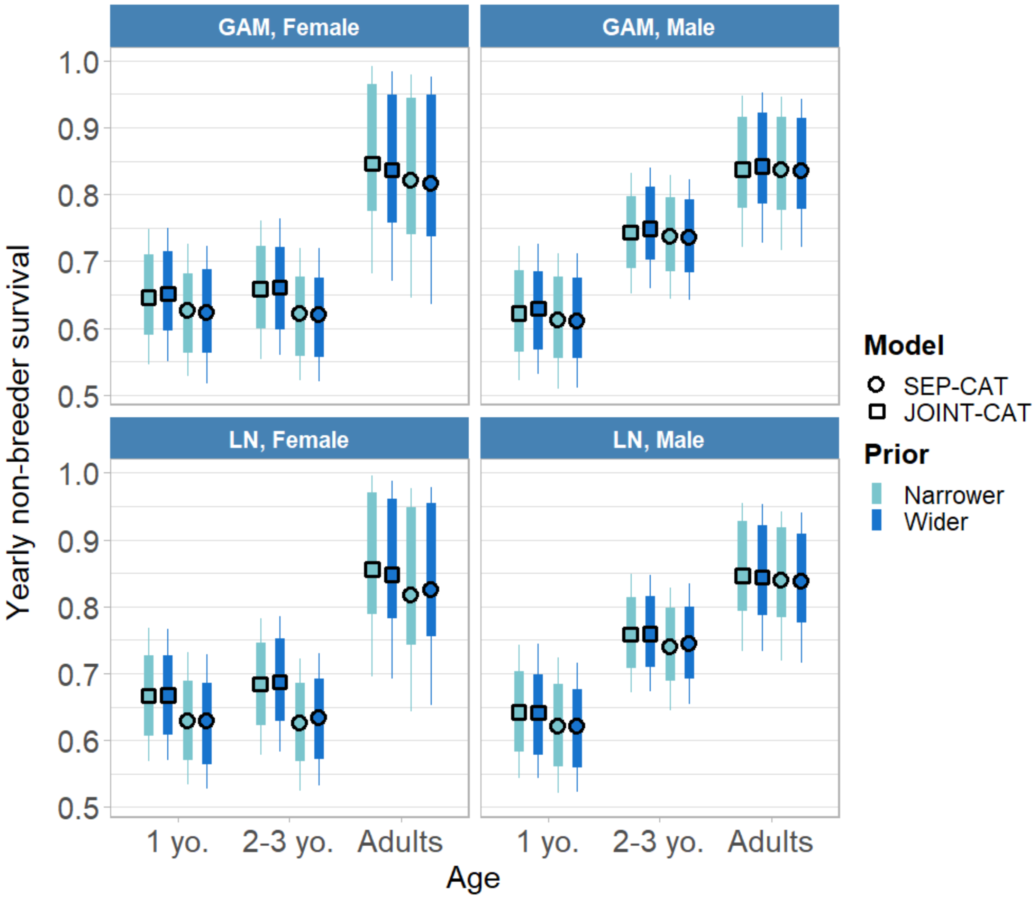

Figure S15. Non-breeder survival posterior estimates of SEP-CAT and JOINT-CAT gamma (GM) and lognormal (LN) models when using narrower priors (i.e., the ones selected in this study) with alternative wider priors. Circles and squares indicate median values. Thick lines indicate the 66% HPDI and fine lines show the 89% HPDI.

The sensitivity analysis reveals very mild differences in the posterior estimates of survival (Figure S15) and average dispersal distance (Figure S16) when using the narrower priors selected in this study and alternative wider priors. Estimates of both survival and dispersal

generally show very limited increases – if any - when using wider prior dispersal kernels with larger expectancies at large distances. Such slight increases appear to occur in both distributions. This suggests that 1) the effect of our selected priors on posterior inference is limited, and 2) the differences found between models and distributions are not explained by our prior dispersal kernel choices.

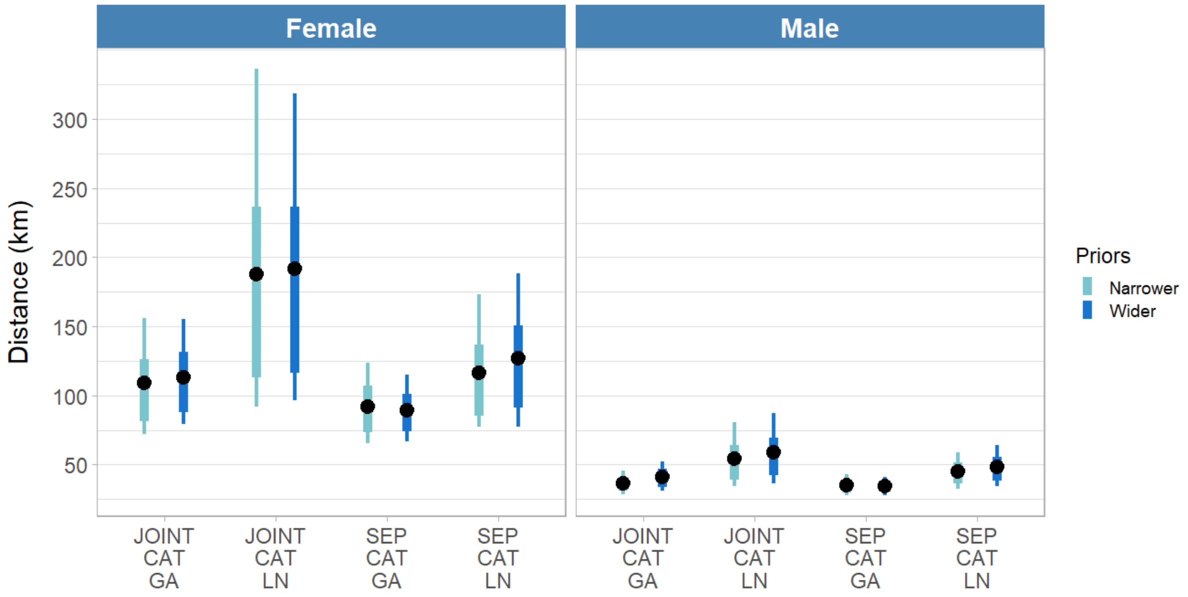

Figure S16. Posterior estimates of average dispersal distance (km) of SEP-CAT and JOINT-CAT models when using narrower priors (i.e., the ones selected in this study) with alternative wider priors. Circles and squares indicate median values. Thick lines indicate the 66% HPDI and fine lines show the 89% HPDI.

References

[1] Gelman, A., Vehtari, A., Simpson, D., Margossian, C.C., Carpenter, B., Yao, Y., Kennedy, L., Gabry, J., Bürkner, P.C., & Modrák, M. (2020). *Bayesian workflow*. arXiv preprint.

[2] Lemoine, N.P. (2019). Moving beyond noninformative priors: why and how to choose weakly informative priors in Bayesian analyses. *Oikos*, 128: 912-928.

[3] Wesner, J.S., & Pomeranz, J.P.F. (2021). Choosing priors in Bayesian ecological models by simulating from the prior predictive distribution. *Ecosphere*, 12(9), e03739.

[4] Hernández-Matías, A., Real, J., Pradel, R., Vincent-Martin, N., Bosca, F., & Cheylan, G. (2010). Determinants of Territorial Recruitment in Bonelli's Eagle (Aquila fasciata) Populations. *The Auk*, 127(1), 173-184.

[5] McElreath, R. Statistical Rethinking: a Bayesian course with examples in R and STAN. Boca Raton, FL: CRC Press.
